# Gene expression network analysis reveals new transcriptional regulators as novel factors in human ischemic cardiomyopathy

**DOI:** 10.1186/s12920-015-0088-y

**Published:** 2015-03-29

**Authors:** Isabel Herrer, Esther Roselló-Lletí, Ana Ortega, Estefanía Tarazón, María Micaela Molina-Navarro, Juan Carlos Triviño, Luis Martínez-Dolz, Luis Almenar, Francisca Lago, Ignacio Sánchez-Lázaro, José Ramón González-Juanatey, Antonio Salvador, Manuel Portolés, Miguel Rivera

**Affiliations:** Cardiocirculatory Unit, Health Research Institute of La Fe University Hospital, Avd de Fernando Abril Martorell, 106, 46026 Valencia, Spain; Genomic Systems, Paterna, Valencia Spain; Heart Failure and Transplantation Unit, Cardiology Department, La Fe University Hospital, Valencia, Spain; Cellular and Molecular Cardiology Unit, Department of Cardiology and Institute of Biomedical Research, University Clinical Hospital, Santiago Compostela, Spain

**Keywords:** Transcriptional regulators, Transcription factor, RNA Sequencing, *BCL3*, *CEBPD*, *CITED2*, *SP100*, Ischemic cardiomyopathy

## Abstract

**Background:**

Ischemic cardiomyopathy (ICM) is characterized by transcriptomic changes that alter cellular processes leading to decreased cardiac output. Because the molecular network of ICM is largely unknown, the aim of this study was to characterize the role of new transcriptional regulators in the molecular mechanisms underlying the responses to ischemia.

**Methods:**

Myocardial tissue explants from ICM patients and control (CNT) subjects were analyzed by RNA-Sequencing (RNA-Seq) and quantitative Real-Time PCR.

**Results:**

Enrichment analysis of the ICM transcriptomic profile allowed the characterization of novel master regulators. We found that the expression of the transcriptional regulators *SP100* (−1.5-fold, p < 0.05), *CITED2* (−3.8-fold, p < 0.05), *CEBPD* (−4.9-fold, p < 0.05) and *BCL3* (−3.3-fold, p < 0.05) were lower in ICM than in CNT. To gain insights into the molecular network defined by the transcription factors, we identified CEBPD, BCL3, and HIF1A target genes in the RNA-Seq datasets. We further characterized the biological processes of the target genes by gene ontology annotation. Our results suggest that CEBPD-inducible genes with roles in the inhibition of apoptosis are downregulated and that BCL3-repressible genes are involved in the regulation of cellular metabolism in ICM. Moreover, our results suggest that *CITED2* downregulation causes increased expression of HIF1A target genes. Functional analysis of HIF1A target genes revealed that hypoxic and stress response genes are activated in ICM. Finally, we found a significant correlation between the mRNA levels of *BCL3* and the mRNA levels of both *CEBPD* (r = 0.73, p < 0.001) and *CITED2* (r = 0.56, p < 0.05). Interestingly, *CITED2* mRNA levels are directly related to ejection fraction (EF) (r = 0.54, p < 0.05).

**Conclusions:**

Our data indicate that changes in the expression of *SP100*, *CITED2, CEBPD,* and *BCL3* affect their transcription regulatory networks, which subsequently alter a number of biological processes in ICM patients. The relationship between *CITED2* mRNA levels and EF emphasizes the importance of this transcription factor in ICM. Moreover, our findings identify new mechanisms used to interpret gene expression changes in ICM and provide valuable resources for further investigation of the molecular basis of human cardiac ischemic response.

**Electronic supplementary material:**

The online version of this article (doi:10.1186/s12920-015-0088-y) contains supplementary material, which is available to authorized users.

## Background

Ischemic cardiomyopathy (ICM) is the most common cause of death in western countries [1]. The factors contributing to ICM are complex. They include microvascular dysfunction, inflammation, disruption of Ca^2+^ homeostasis, and activation of apoptosis [2,3]. In addition, studies have indicated that a number of fetal and immediate-early genes, including those encoding proteins involved in signal transduction and energy metabolism, are deregulated in the ischemic heart [4,5]. These altered processes are associated with a specific gene expression pattern, or transcriptional signature. The transcriptional signature of ICM is thought to arise from ischemic injury or other types of stress stimulus, ultimately resulting in heart failure (HF). Although several global gene expression studies have been carried out in ICM [6], the molecular mechanisms that coordinate the transcriptional profile in ICM are not completely understood.

Gene expression is determined by specific sets of transcription factors (TFs) and by a particular organization, or chromatin structure, of the genome. Several TFs have been implicated in ICM [7]. Specifically, our group identified changes in the protein levels of TFs including nuclear factor of activated T cells 1 (NFAT1), GATA binding protein 4 (GATA4), and nuclear myocyte enhancer factor 2C (MEF2C) in ICM patients [8]. These DNA binding factors are involved in the Ca^2+^ signaling and may also participate in apoptosis, highlighting the importance of these processes in ICM. Other TFs implicated in ICM include the homeobox protein CSX/NKX2-5 [9], which participates in the transcriptional regulation of fetal and early-stage genes, the NF-kB pathway activator protein [10], STAT-3 [11], and AP-1 [12]. Despite these findings, the regulatory mechanisms underlying the disruption of essential biological processes in ICM, such as angiogenesis [13] and cellular metabolism, remain to be elucidated.

A traditional approach for identifying the underlying causes of a specific disease is to look for genes that are differentially expressed in disease samples and the appropriate control (CNT) samples. Microarray-based expression profiling has been widely used for this purpose. In addition, gene expression profiling has been used to identify TFs involved in biological processes [14] and in diseases such as dilated cardiomyopathy [15]. There are no genome-wide expression analysis-based studies related to TFs in ICM. RNA sequencing (RNA-Seq) has recently emerged as a precise and sensitive method for mapping and quantifying RNA transcripts [16]. This technology can potentially be used to identify novel transcriptional regulators involved in the molecular mechanisms controlling ICM. Therefore, we aimed to identify novel transcriptional regulators with roles in in ICM development and to characterize their target genes in order to provide novel insights into the mechanisms involved in the responses to ischemia.

## Methods

### Ethical approval

This study was approved by the Biomedical Investigation Ethics Committee of La Fe University Hospital of Valencia, Spain. This work was performed in accordance with the guidelines of the Declaration of Helsinki [17]. Informed written consent was obtained from each patient prior to tissue collection.

### Tissue collection

Left ventricular (LV) tissue samples were collected from human hearts of 13 and 19 patients with ICM undergoing cardiac transplantation and subsequently used in RNA-Seq and quantitative Real-Time PCR (qRT-PCR), respectively. The clinical characteristics of the patients are shown in Table 1. Clinical history, electrocardiography, hemodynamic studies, Doppler echocardiography, and coronary angiography data were available. All patients were functionally classified according to the New York Heart Association (NYHA) criteria and were receiving medical treatment following the guidelines of the European Society of Cardiology [18]. ICM was diagnosed on the basis of the clinical history, Doppler echocardiography, and coronary angiography data.

**Table 1:** **Clinical characteristics of patients with ischemic cardiomyopathy**

	**RNA-Seq**	**qRT-PCR**
	**ICM (n = 13)**	**ICM (n = 19)**
**Age (years)**	54 ± 7	53 ± 7
**Gender male (%)**	100	85
**NYHA class**	3.5 ± 0.4	3.1 ± 0.9
**BMI (kg/m** ^**2**^ **)**	26 ± 4	27 ± 3
**Hemoglobin (mg/mL)**	14 ± 3	13 ± 3
**Hematocrit (%)**	41 ± 6	38 ± 8
**Total cholesterol (mg/dL)**	162 ± 41	167 ± 33
**Prior hypertension (%)**	30	31
**Prior smoking (%)**	84	58
**Prior diabetes mellitus (%)**	38	36
**EF (%)**	24 ± 4	23 ± 8
**FS (%)**	13 ± 2	12 ± 4
**LVESD (mm)**	55 ± 7	53 ± 7
**LVEDD (mm)**	64 ± 7	60 ± 6
**LV mass index (g/cm** ^**2**^ **)**	139 ± 36	130 ± 34

Data are showed as the mean value ± SD or % of subjects. ICM, ischemic cardiomyopathy; NYHA, New York Heart Association; BMI, body mass index; EF, ejection fraction; FS, fractional shortening; LVESD, left ventricular end-systolic diameter; LVEDD, left ventricular end-diastolic diameter; LV mass index, left ventricular mass index.

CNT LV samples were obtained from the hearts of six (RNA-Seq) or seven (qRT-PCR) healthy donors whose hearts could not be transplanted due to surgical reasons or blood type incompatibility. The cause of death in these individuals was cerebrovascular or motor vehicle accident. All donors had normal LV function and had no history of myocardial disease or active infection at the time of transplantation.

Fresh transmural samples were recovered from near the apex of the left ventricle at the time of transplantation. Tissue samples were maintained in 0.9% NaCl at 4°C for a maximum of 6 hours from the time of coronary circulation loss and then frozen at −80°C until RNA extraction.

### RNA extraction

Heart samples were homogenized with TRIzol® Reagent in a TissueLyser LT (Qiagen; UK). All RNA extractions were performed using a PureLink® RNA Mini Kit (AmbionLife Technologies; CA, USA), according to the manufacturer’s instructions. RNA was quantified using a NanoDrop1000 spectrophotometer (Thermo Fisher Scientific; UK) and the purity and integrity of the RNA samples were measured using an Agilent 2100 Bioanalyzer with the RNA 6000 NanoLabChip kit (Agilent Technologies; Spain). All samples displayed a 260/280 nm absorbance ratio greater than 2.0 and RNA integrity numbers ≥ 9.

### RNA-Seq

PolyA-RNA was isolated from 25 μg of total RNA using the MicroPoly(A) Purist kit (Ambion, USA). Total PolyA-RNA was used to generate whole transcriptome libraries for sequencing on the SOLiD 5500XL platform following the manufacturer’s recommendations (Life Technologies; CA, USA). Amplified cDNA quality was analyzed using the Bioanalyzer 2100 DNA 1000 kit (Agilent Technologies; Spain) and quantified using the Qubit 2.0 Fluorometer (Invitrogen; UK). Whole transcriptome libraries were used to make SOLiD templated beads following the SOLiD templated bead preparation guide. This protocol consisted of an RNA enrichment and chemical modification step, followed by a clonal amplification step. Bead quality was estimated based on work flow analysis parameters. The samples were sequenced using the 50625 paired-end protocol, generating 115 nt sequences consisting of 75 nt plus 35 nt (Paired-End) + 5 nt (Barcode). Quality data was measured using software parameters of the SOLiD Experimental System.

### Computational analysis of RNA-Seq data

The initial whole transcriptome paired-end reads obtained from sequencing were mapped against the latest version of the human genome (version GRchr37/hg19) using the Life Technologies mapping algorithm (http://www.lifetechnologies.com/), version 1.3. For both forward and reverse reads, the seed was the first 25 nucleotides with a maximum of 2 mismatches allowed. Additional file 1: Table S1 describes the main statistical parameters of the mapping analysis. The aligned records were reported in BAM/SAM format [19]. Insufficient quality reads (phred score < 10) were eliminated using Picard Tools software, version 1.83 (http://picard.sourceforge.net/). Gene prediction was estimated using Ensembl ID and the Cufflinks method for *de novo* assembly [20]. After alignment the read counts or gene expression levels were calculated using HTSeq software, version 0.5.4p3 (http://www-huber.embl.de/users/anders/HTSeq/). Differential expression analysis between conditions was performed using the edgeR method, version 3.2.4 [21]. This method uses a Poisson distribution to model genic read counts following normalization based on size factors and variance; therefore, this software allows normalization of RNA-Seq data based on sequencing depth, GC content, and gene length for analysis of differential expression. We selected differentially expressed genes with a *p*-value < 0.05 and a fold change of at least 1.5. Primary RNA-Seq data were submitted to the public database Gene Expression Omnibus (GEO) repository, and the accession number to the data file is GSE55296 (http://www.ncbi.nlm.nih.gov/geo/query/acc.cgi?acc=GSE55296).

Finally, we assessed the technical variation by Pearson correlation analysis using the R-statistical software, version 3.0.3 (http://www.r-project.org).

### Principal component analysis

To classify heart samples, a principal component analysis (PCA) was performed with the RNA-Seq data. A scatter plot was produced in order to visualize the differences between the sample sets based on each sample’s gene expression profile. This analysis was performed using R-statistical software, version 3.0.3 (http://www.r-project.org).

### Gene set enrichment analysis

To identify over-represented TFs from RNA-Seq data, we used a web-based interactive application called ChIP Enrichment Analysis (ChEA) [22]. The ChEA database contains data from genome-wide ChIP studies and therefore takes into consideration the chromatin state of the cell. TFs showing a p-*value* < 0.05 were considered significant.

### qRT-PCR

One microgram of RNA was reverse-transcribed to cDNA using the M-MLV enzyme (Invitrogen, UK). qRT-PCR was performed in duplicate using the TaqMan protocol in a ViiA7 Fast Real-Time PCR System according to the manufacturer’s instructions (Applied Biosystems; USA). The following TaqMan probes were designed and obtained from Applied Biosystems: SP100 nuclear antigen (*SP100) (Hs00162109_m1*), Cbp/p300-interacting transactivator 2 (*CITED2) (Hs00366696_m1),* CCAAT/enhancer binding protein delta (*CEBPD) (Hs00270931_s1)* and B-cell CLL/lymphoma 3 *(BCL3) (Hs00180403_m1).* The housekeeping genes *GAPDH (Hs99999905_m1), PGK1* (*Hs99999906_m1),* and *TFRC* (*Hs00951083_m1)* were used as reference genes. ΔΔCt-based fold change calculations were used to determine relative transcript quantity [23]. Individual fold changes were calculated comparing each ICM ∆Ct value with the corresponding CNT pooling ∆Ct values.

### TF target genes prediction

To decipher the transcriptional regulatory networks, we identified the TF target genes that were differentially expressed between ICM patients and CNT individuals. TF target gene prediction was carried out using the transcriptional regulatory element database (TRED) (http://rulai.cshl.edu/TRED), TFactS (http://www.tfacts.org*), and the* ChIP-X database, a component of the ChEA *software.* All three informatics tools encompass the prediction of TF regulation based on TF binding motifs and experimental evidence.

### TF target genes functional annotation

Functional annotation analysis of differentially expressed genes was performed using the Database for Annotation, Visualization and Integrated Discovery (DAVID, version 6.7). Gene ontology (GO) terms that had a *p*-value < 0.05 were selected [24].

### Statistics

Data are expressed as the mean ± standard deviation (SD). The Kolmogorov-Smirnov test was applied to evaluate the data distribution. Clinical characteristics were compared using Student’s *t*-test for continuous variables and Fisher’s exact test for discrete variables. Significant mean differences in mRNA levels between groups were determined using Student’s *t*-test. Pearson’s correlation coefficient was calculated to analyze the association between mRNA levels and the relationship between mRNA expression levels and clinical parameters. A *p-*value < 0.05 was considered significant. Statistical analysis was performed using the Statistical Package for Social Sciences, version 20.0 (IBM SPSS Inc., Chicago. IL, USA) and GraphPad Prism software version 6.0 (http://www.graphpad.com).

## Results

### Clinical characteristics of patients

We analyzed by RNA-Seq a total of 19 LV tissue samples corresponding to hearts from 13 ICM patients undergoing cardiac transplantation and six non-diseased CNT donors. ICM patients were 100% male, and were a mean age of 54 ± 7 years. All of them had a NYHA functional classification of III–IV and previously had been diagnosed with significant comorbidities, including hypertension and hypercholesterolemia (Table 1). The CNT group comprised 83% men and the mean age was 53 ± 10 years.

We used a greater sample size for qRT-PCR validation of up to 26 LV samples corresponding to hearts from 19 ICM patients and seven non-diseased CNT donors. ICM patients were 85% male, and were a mean age of 53 ± 7 years. Their clinical characteristics are shown as the mean value ± SD in Table 1. No significant differences were found in clinical parameters between the two ICM groups. The CNT group comprised 75% men and the mean age was 51 ± 9 years.

### RNA-Seq results

**Figure 1:**
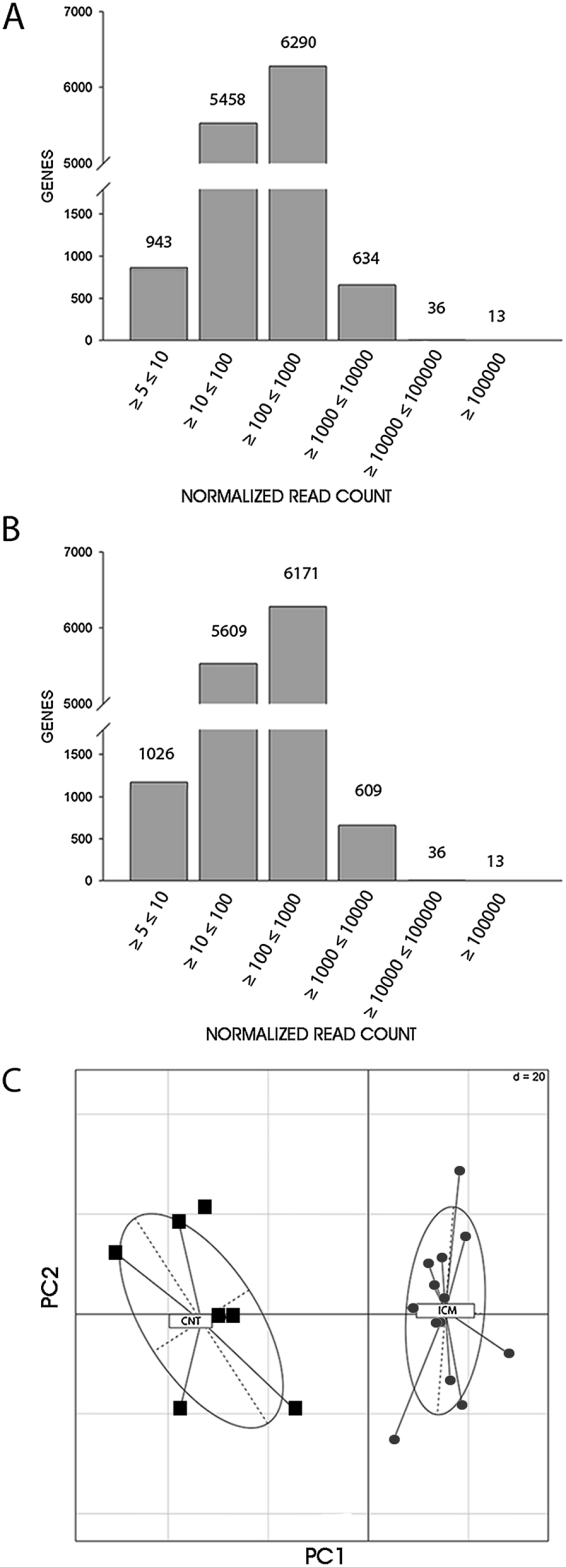
**Transcriptomic profiling of heart samples by RNA-Seq.** The number of genes and their relative expression levels represented by transcript normalized read counts in controls **(A)** and ICM patients **(B)**. **(C)** Principal Component Analysis of heart samples were cluster on the basis of their gene expression profile. Samples are represented by points at CNT for controls and ICM for patients. Proportions of variances showed that 0.95% of the differences among the sample groups could be explained by PCA component 1 (PC1) and 0.233% by PCA component 2 (PC2).

To investigate the transcriptomic changes accompanying ICM, we performed a large-scale expression screen using RNA-Seq technology. Nineteen heart samples were used for the analysis (ICM, n = 13 and CNT, n = 6). Primary RNA-Seq data were submitted to the public database Gene Expression Omnibus (GEO) repository [accession number GSE-55296]. Results of pairwise Pearson-correlations between samples showed no significant differences (Additional file 2: Figure S1). We applied a threshold of 0.85. Since inclusion of transcripts expressed at very low levels increases the risk of false discovery, a minimum of five normalized transcript read counts was used as the cut-off point. Transcripts from 13,374 genes were identified in CNT hearts and from 13,464 genes were detected in ICM tissue by RNA-Seq. For transcript identification we used Ensembl ID information and the Cufflinks method for the unknown genes. Expressed genes were divided into groups according to their relative expression levels (Figure 1A-B). We found that 943 and 1,026 genes showed the lowest levels of expression, represented by five and ten read counts in CNT and ICM, respectively. While the majority of genes in CNT (88%; 11,748/13,374) and in ICM (87%; 11,780/13,464) displayed between 10 and 1000 read counts, only 13 genes in CNT hearts and in ICM samples were highly expressed, with ≥100,000 read counts (Figure 1A-B). The most abundantly transcribed genes were *MT-ND4* (NADH dehydrogenase subunit 4, a mitochondrial-encoded gene), with a read count of 2,422,108, in CNT and *MT-COI* (cytochrome c oxidase subunit I, a mitochondrial-encoded gene) in ICM, with a read count of 2,170,339. Most prevalent among the genes with read counts of approximately 100,000 in both types of samples were those encoding cytoskeletal components, including *MYL2, ACTLC1,* or *DES*.

Significance analysis of the RNA-Seq results revealed a total of 1,334 genes that were differentially expressed in ICM patients *vs* CNT; 649 genes were upregulated (≥1.5 fold, p < 0.05) and 685 genes were downregulated (≤1.5 fold, p < 0.05). These genes encompass the ICM transcriptome signature. In order to validate these genetic differences, clustering analysis of the samples was performed by PCA with the differentially genes. When PCA is applied to data, samples with similar trends in their gene expression profiles tend to cluster close together in the plot. PCA demonstrated that the gene expression profiles showed a clear distinction between ICM patients and CNT group (Figure 1C).

### TF enrichment analysis

**Figure 2:**
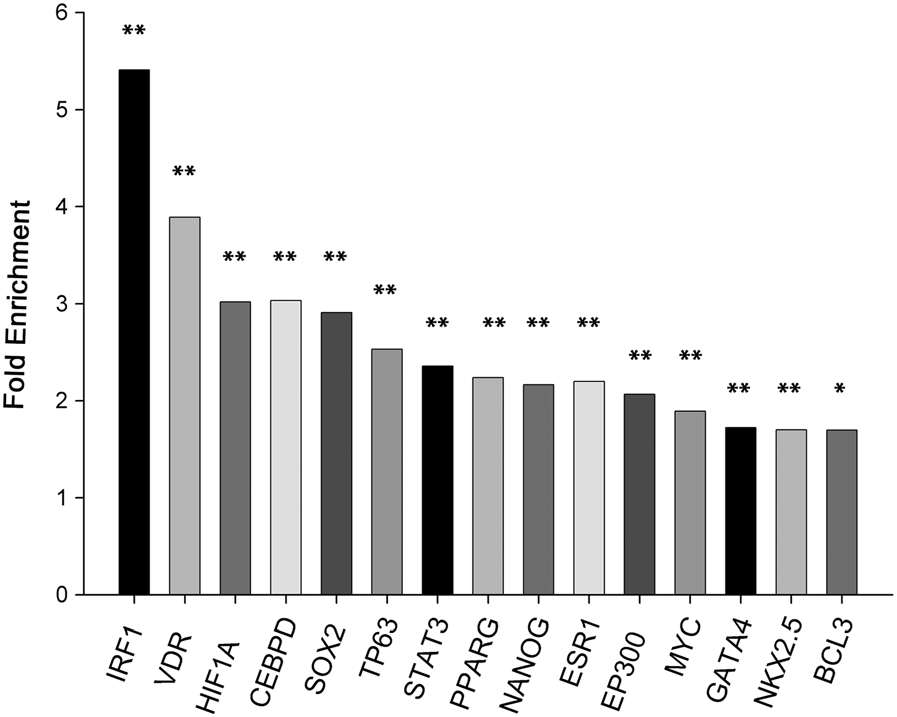
**Transcription factor (TF) enrichment analysis.** Relevant TFs identified using the ChEA database and based on the RNA-Seq genetic profile. The graph shows the official name and the fold enrichment of each TF in ICM *vs* CNT. Fold enrichment was calculated by an algorithm considering target genes of our list/target genes ChIP-X database relation. All comparisons were statistically significant (*p < 0.01, **p < 0.001).

In order to identify key TFs mediating the differential expression of genes in ICM, the transcriptional signature was analyzed in ChEA database containing 94% of these genes. Using ChEA enrichment analysis, we identified TFs associated with ICM at a *p*-value < 0.05, calculated by Fisher’s exact test (Additional file 3: Table S2). Figure 2 shows relevant TFs with a *p*-value < 0.01. These TFs include those previously associated with ICM, including GATA4 (1.7-fold enrichment), NKX2.5 (1.7-fold enrichment), STAT3 (2.4-fold enrichment), and EP300 (2-fold enrichment) [8,9,11,25], demonstrating the reliability of this database. In addition, TFs such as ESR1 (2.2-fold enrichment), and the pluripotency markers SOX2 (2.9-fold enrichment) and NANOG (2-fold enrichment), have not been previously implicated in human ICM. HIF1A (3.02-fold enrichment) was significantly over-represented in ICM, as were CEBPD (3-fold enrichment), and BCL3 (1.7-fold enrichment) (Figure 2). Furthermore, RNA-Seq data showed that gene expression levels of *CEBPD, BCL3,* and the HIF1A repressor, *CITED2,* significantly decreased in ICM patients (Table 2).

**Table 2:** **Selected genes analyzed by RNA-Seq and used for qRT-PCR validation**

**Gene ID**	**Gene symbol**	**Fold change (ICM** ***vs*** **CNT)**	**p value**
ENSG00000067066	*SP100*	−1.8994	0.0005
ENSG00000164442	*CITED2*	−1.5631	0.0215
ENSG00000221869	*CEBPD*	−3.2139	2.20E-09
ENSG00000069399	*BCL3*	−1.9970	1.38E-05

Not all TFs identified in the enrichment analysis were differentially expressed according our RNA-Seq results. Since protein levels, post-transcriptional modifications, and other factors that affect TF regulatory function can also influence the activities of target genes, it is not surprising to find overrepresentation of a specific TF signaling pathway without a change in the gene expression of the TF.

On the other hand, the TFs NFAT1 and MEF2C, already found in ICM patients in previously studies [8], are not included in the ChIP-X database. Because ChIP studies that profile the binding of NFAT1 and MEF2C are not available, these proteins were not identified in the TF enrichment analysis.

### qRT-PCR analysis

Next, we used qRT-PCR to validate the RNA-seq data indicating differences between ICM and CNT samples in the mRNA levels of *SP100, CITED2, CEBPD,* and *BCL3,* four transcriptional regulators that have not been implicated previously in ICM*. SP100* encodes a transcriptional coactivator/corepressor that regulates DNA-binding by other TFs, and *CITED2*, *CEBPD* and *BCL3* encode TF that are closely involved in stress or hypoxia responses [26]. Given the importance of these processes in disease development, we considered it interesting to further study their regulatory mechanisms. As a result, we validated the expression levels of these genes.

**Figure 3:**
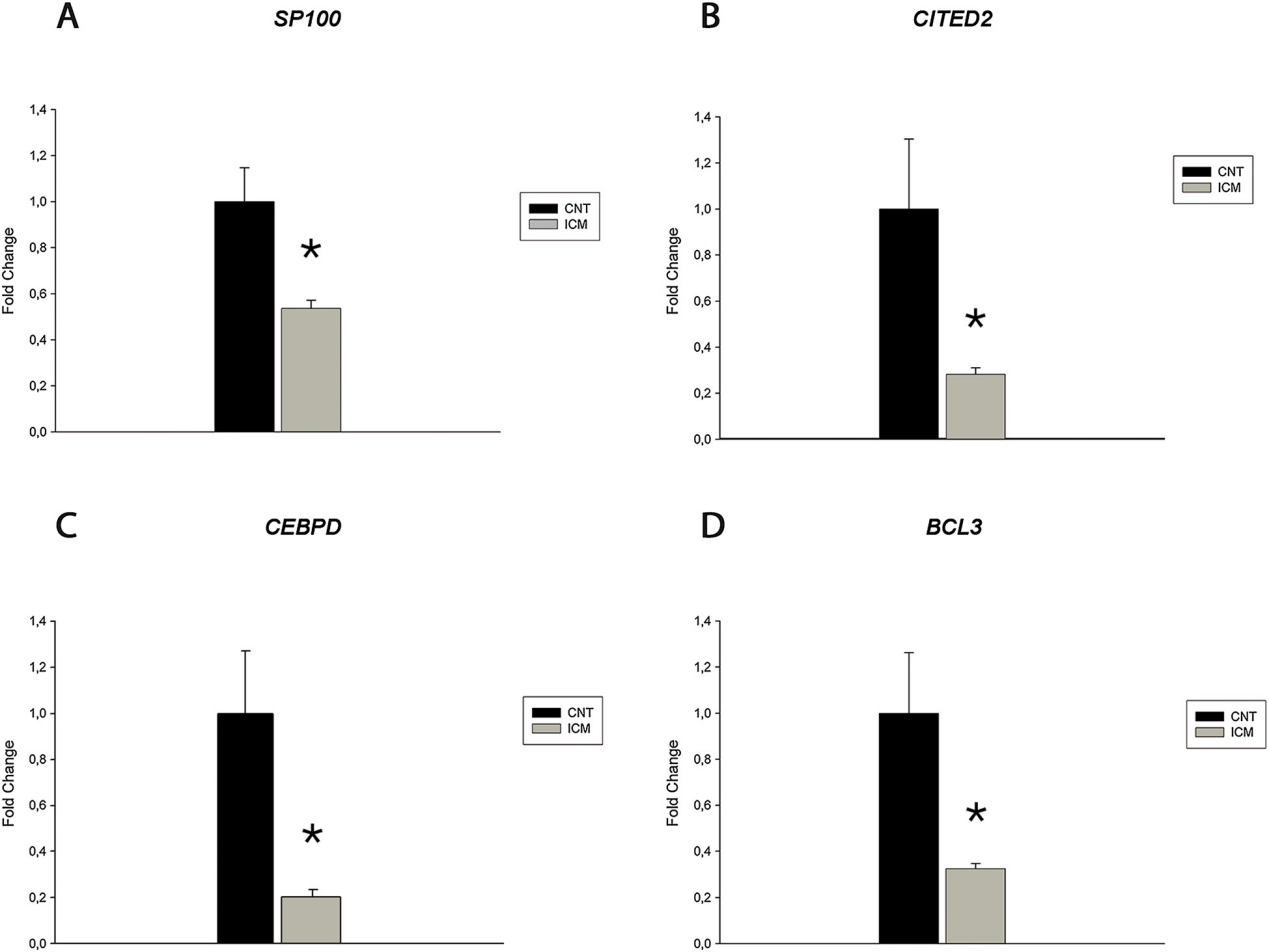
**Expression levels analysis of transcriptional regulators genes in ICM** ***vs*** **CNT hearts.** The relative differential expression in ICM comparing to CNT was quantified by qRT-PCR using the ΔΔCt method for *SP100* **(A)**, *CITED2* **(B)**, *CEBPD* **(C)**, and *BCL3* **(D)**. *GAPDH, PGK1,* and *TFRC* were used to normalize. Results were considered statistically significant at *p < 0.05. Bars represent the mean ± SEM.

**Figure 4:**
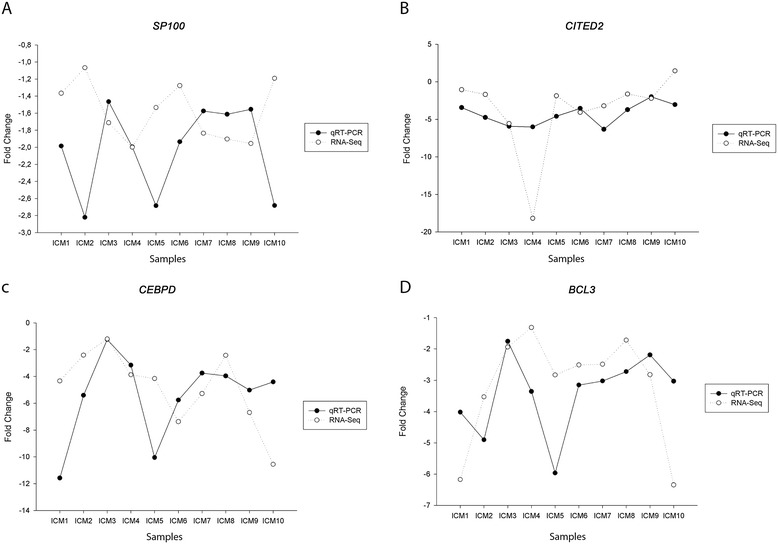
**qRT-PCR** ***vs*** **RNA-Seq data comparison.** Individual ICM *vs* CNT fold change data obtained by qRT-PCR and RNA-Seq (Y-axis) were represented for each patient (X- axis) in *SP100* **(A)**, *CITED2* **(B)**, *CEBPD* **(C)**, and *BCL3* **(D)**. Slight differences are visualized as the gaps between the points of the two spline curve lines in the scatter-plot.

qRT-PCR confirmed lower expression of *SP100* (−1.5-fold, p < 0.05) in ICM than in CNT (Figure 3). In addition, the expression of the TF genes *CITED2* (−3.8-fold, p < 0.05), *CEBPD* (−4.9-fold, p < 0.05) and *BCL3* (−3.3-fold, p < 0.05) were significantly lower in ICM *vs* CNT hearts (Figure 3). Further verification of fold change values for these genes was performed by comparing qRT-PCR and RNA-Seq data from the same 10 patient samples. The direction and degree of fold changes were similar in all cases for RNA-Seq and qRT-PCR. For most of the genes analyzed there was a good correlation between both techniques, although, generally, greater fold change differences were detected for the downregulated genes by qRT-PCR (Figure 4).

### TF target genes analysis

To gain insights into alterations in the gene regulatory network in ICM patients, we identified downstream responsive genes of CEBPD, BCL3, and HIF1A and then compared RNA-Seq expression levels of these genes between ICM and CNT LV samples. We performed an integrative bioinformatic approach using three prediction databases: TRED, TFactS, and ChEA. The results from the database analyses were complemented with results from a previous study by Yang et al. [27], which determined the transcriptomic signature of cardiomyocytes overexpressing *BCL3*. Although the protein encoded by *SP100* binds to chromatin and regulates gene expression [28], is not defined as a TF so we did not use it in the following analysis.

Results from the target predictions showed that a total of 25 CEBPD target genes were downregulated in the cardiac tissue of ICM patients, and that these targets encompassed members of the TGFβ family, including *TGFBR2* and *BMPR1B.* In addition, 70 BCL3 target genes were downregulated in ICM patients, including genes encoding TFs (*STAT2, FOSL2, GABPR2*), cytokines (*CXCL1, CXCL10*), and growth factor receptors (*TGFBR3*). CITED2 is a TF without typical DNA binding domains, and a well-known corepressor of HIF1A transcriptional activation [29]; so we further studied the potential function of HIF1A and CITED2 through changes in the expression of HIF1A target genes. We found that 17 HIF1A target genes were upregulated in ICM patients, including well-known hypoxic genes such as *EGLN3* and *IGFBP3* [30]. All target genes of CEBPD, BCL3, and HIF1A are shown in Additional file 4: Table S3.

We next analyzed the functional downstream effects of the differentially expressed genes using the DAVID software for GO annotation. A significant enrichment (*p* < 0.05) indicates specific biological processes that are affected when the corresponding genes are altered. GO results indicated that the downregulated CEBPD target genes are mainly involved in activating apoptosis and that the BCL3 target genes are involved in energy metabolism, including the regulation of responses to glucose levels (Table 3). Finally, genes regulated by HIF1A participate in hypoxic and stress responses (Table 3). Additional GO terms related to biological processes and significantly represented by genes downstream of CEBPD, BCL3 and HIF1A are shown in Additional file 5: Table S4.

**Table 3:** **Identification and functional annotation analysis of TF target genes**

**FT**	**GO**	**p-value**	**Genes**
CEBPD	System development	5.00E-05	*CITED2, RGMA, BMPR1B, CALCRL, FGF7, IL1B, IRX3, LRRC4C, RXFP2, TGFBR2, TNFRSF11A*
CEBPD	Negative regulation of apoptosis	1.10E-02	*CITED2, RIPK2, IL1B, RXFP2*
CEBPD	Negative regulation of programmed cell death	1.20E-02	*CITED2, RIPK2, IL1B, RXFP2*
CEBPD	Blood vessel morphogenesis	3.20E-02	*CTH, IL1B, RXFP2, TGFBR2*
CEBPD	Negative regulation of cell proliferation	1.20E-02	*CITED2, TGFBR2, IL1B*
BCL3	Regulation of metabolic process	4.80E-04	*PFKFB2, ABCA1, ATPIF1, FOSL2, GABPB2, SP110, TSPYL2, ATF7, A2M, ANKRD1, CALCRL, GDF7, HR, MLYCD, MYD88, NR4A1, NR4A3, PER1, PIM1, PDCD4, PSMB9, STAT2, SOAT1, TXNIP, TGFBR2, TGFBR3, UBB, UBE2L6, ETS2*
BCL3	Regulation of nitrogen compound metabolic process	6.30E-03	*ABCA1, ATPIF1, FOSL2, GABPB2, SP110, TSPYL2, ATF7, ANKRD1, CALCRL, GDF7, HR, MYD88, NR4A1, NR4A3, PER1, PIM1, PDCD4, STAT2, TXNIP, TGFBR3, UBB, ETS2*
BCL3	Response to glucose stimulus	1.60E-02	*PFKFB2, TXNIP, TGFBR2*
BLC3	Response to hexose stimulus	1.80E-02	*PFKFB2, TXNIP, TGFBR2*
BCL3	Response to monosaccharide stimulus	1.80E-02	*PFKFB2, TXNIP, TGFBR2*
CITED2/HIF1A	Response to hypoxia	9.80E-06	*ANGPTL4, EGLN3, NOL3, KCNA5*
CITED2/HIF1A	Response to oxygen levels	1.20E-05	*ANGPTL4, EGLN3, NOL3, KCNA5*
CITED2/HIF1A	Response to stress	9.20E-04	*ANGPTL4, CCND1, EGLN3, MIF, NOL3, KCNA5, TFF3*
CITED2/HIF1A	Regulation of cell proliferation	8.00E-03	*CCND1, DPT, EGLN3, ADM, IGFBP3*

### Relationship between gene expression levels and LV function parameters

**Figure 5:**
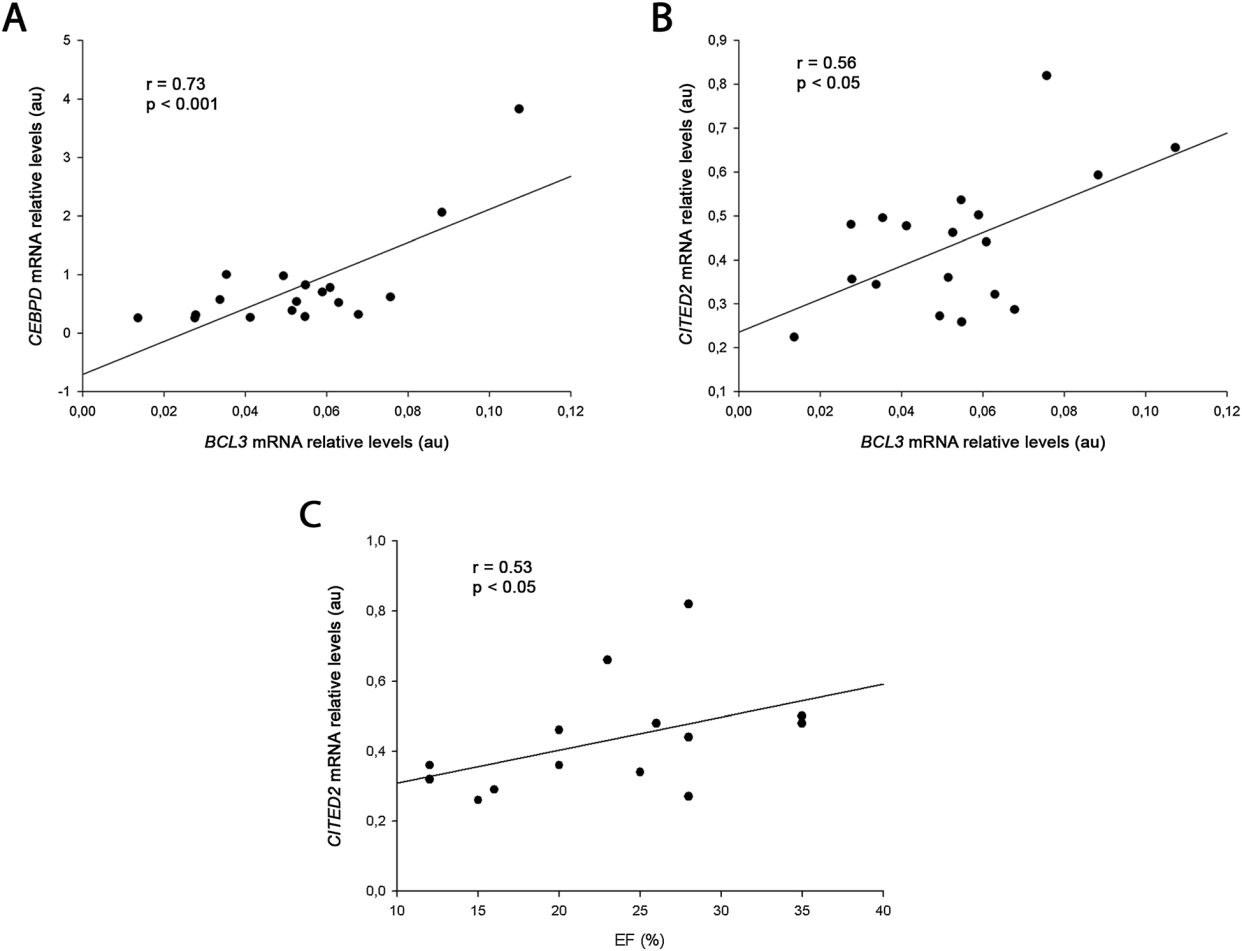
**Relationship between expression levels of transcription factors and left ventricular function parameter. (A)** Correlation between *BCL3 vs CEBPD* mRNA relative levels (n = 18). **(B)** Correlation between *BCL3 vs CITED2* mRNA relative levels (n = 18). **(C)** Correlation between *CITED2* mRNA relative levels and ejection fraction (n = 14). Arbitrary units (au).

In order to determine a potential association between the dysregulated genes in ICM, we determined whether there was some relation between changes in gene expression levels. The results showed that *BCL3* mRNA levels were significantly correlated with *CEBPD* (r = 0.73, p < 0.001) and *CITED2* (r = 0.56, p < 0.05) mRNA levels (Figure 5A-B). We also investigated the association between the mRNA expression levels and the clinical parameters of the patients. Ejection fraction (EF) represents the fraction of blood that leaves the left and right ventricles when the heart contracts. The EF clinical data from the ICM patients were available for 14 of the samples used in qRT-PCR analysis. We found a direct correlation between *CITED2* mRNA expression levels and LV EF (r = 0.54, p < 0.05) (Figure 5C).

## Discussion

The regulation of gene expression is dependent on the expression profiles of specific TFs, and also involves chromatin remodeling complexes that regulate the access of TF to DNA sequences. These mechanisms represent a key node in maintaining cell homeostasis, and the dysregulation of these interactions has been associated with diseases, including ICM [31]. While TFs involved in altered Ca^2+^ homeostasis, inflammation, and apoptosis disruption have been associated with ICM [2], little is known about the specific signaling response that is affected. The role of TFs in orchestrating other important processes, such as stress and hypoxic responses, is also not clearly understood. Moreover, although previous microarray studies have identified ICM specific gene expression profiles [6,32], the regulatory mechanism underlying these profiles has not yet been determined. We hypothesized that novel regulatory factors could function to coordinate the gene expression signatures involved in the etiology or development of ICM.

In this work, we have described the spectrum of genes expressed in CNT and ICM heart tissue, identified by RNA-Seq. Transcriptomic analysis indicated that genes that encode components of the respiratory electron transport are those that are among most abundantly expressed. The analysis also indicated that cytoskeleton genes are highly expressed, demonstrating the significant abundance of their components in the heart. The differential gene expression profile between ICM and CNT LV samples was used to perform an enrichment analysis to identify relevant TFs. Enrichment analysis of RNA-Seq results identified TFs previously found to be involved in ICM, including EP300, GATA4 [8], STAT-3 [11] and NKX2-5 [33]. Moreover, the identification of ESR1 as a regulatory factor in human ICM in this study correlates with the association of specific polymorphisms in the *ESR1* gene to coronary heart disease [34]. In addition, evidence of the pluripotency of the TFs MYC, NANOG, and SOX2 in ICM suggests that they may participate in the fetal gene re-expression program in ICM [35].

We next focused our study on analyzing the expression levels of the TFs genes *CITED2, CEBPD,* and *BCL3,* which are involved in the regulation of the stress or hypoxic response, and on the *SP100* gene, which encodes a heterochromatin binding factor. qRT-PCR analysis validated the downregulation of these transcriptional regulators in ICM.

The *SP100* gene encodes a nuclear body component that has an important role in chromatin-mediated gene regulation [36]. It has been proposed that suppression of *SP100* activates cell immortalization, leading to genomic instability and cytoplasmic sequestration of p53 [37]. Interestingly, ICM hearts are characterized by presenting DNA fragmentation [38], and recently it has been shown that cytosolic sequestration of p53 facilitates mitochondrial dysfunction and HF in mice [39]. These data suggest that decreased *SP100* expression in ICM may be associated with these molecular heart alterations.

To gain insights into the molecular network of the differentially expressed TFs, we studied their potential roles in ICM by characterizing changes in the expression of their putative target genes and alterations in associated processes. Bioinformatics analysis of the RNA-Seq data identified target genes of CEBPD, BCL3, and CITED2 that are potentially involved in ICM.

Twenty-five CEBPD target genes were downregulated in ICM patients, some of which are implicated in apoptosis via downregulation of *RIPK2* and *RXFP2* [40]. Many of the 70 BCL3 target genes that are downregulated in ICM patients are involved in the regulation of energetic biological processes and in the inhibition of the glucose response [41]. This suggests that *BCL3* downregulation may be the underlying cause of the metabolic disruption of the carbohydrate responses in these patients. These data are consistent with a previous study suggesting cooperation between BCL3 and PCG-1α in the coordination of inflammation and energy metabolism in the heart [31]. It should be noted, however, that our RNA-Seq data did not detect significant differences in *PCG-1α* expression between ICM and CNT samples. So the physiological mechanism may not be completely disrupted by the altered BCL3 response in the disease. BCL3 is an IkB protein that associates tightly with p50 or p52 homodimers as an activator component of the NF-kB pathway [42,43], and it has been shown that NF-kB activation is characteristic of failing myocardium [44,45]. Functional characterization of genes downstream of *BCL3* downstream genes did not identify the NF-kB pathway. Activation of the NF-kB pathway in ICM patients may be mediated by mechanisms that are independent of BCL3.

**Figure 6:**
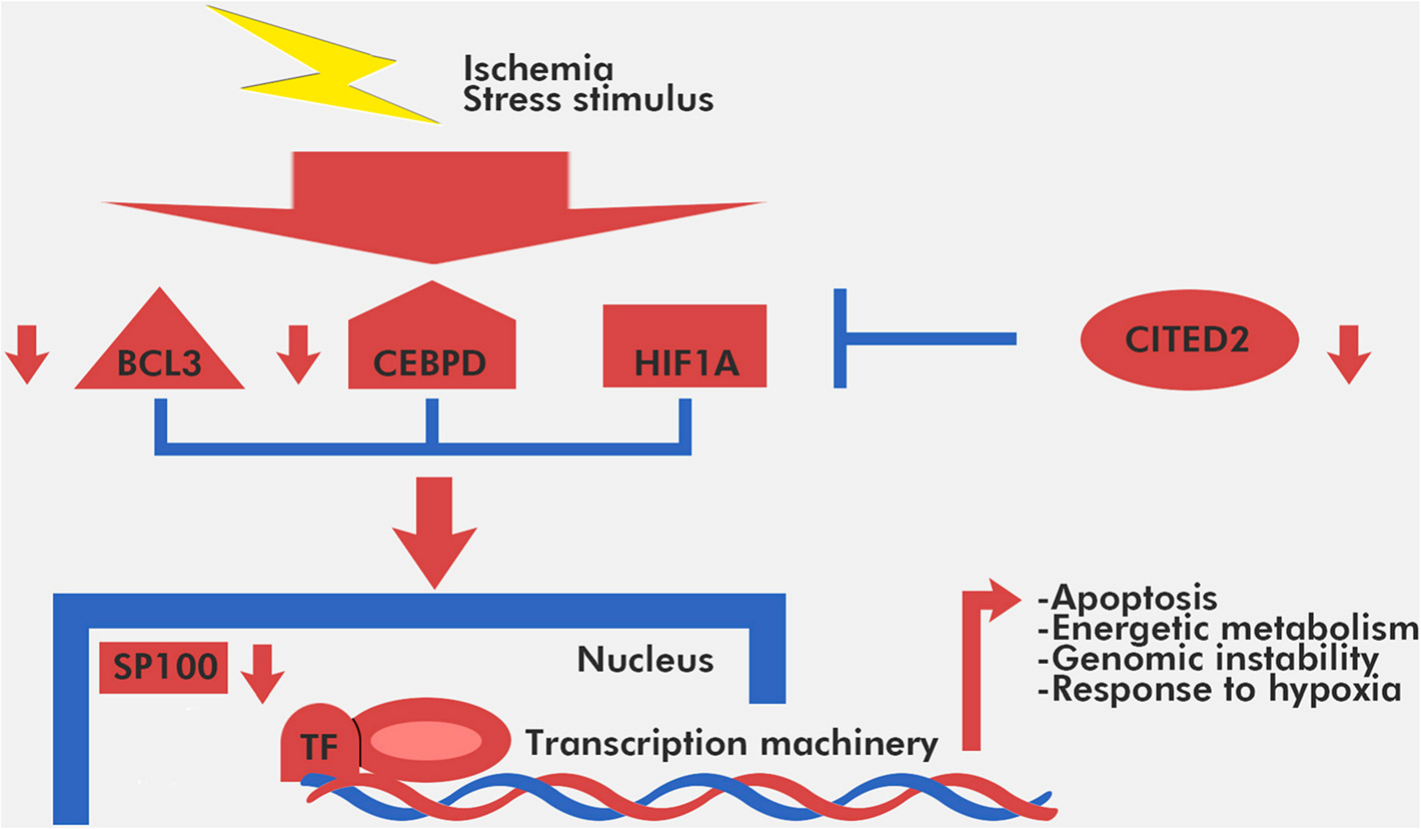
**Hypothetical model of the transcriptional regulatory network in ICM patients.** Expression of the TFs*, CITED2, CEBPD* and *BCL3*, and *SP100* is downregulated under conditions of hypoxia and other stress stimuli, which in turn modifies the expression of their target gene networks. These changes ultimately result in alterations in cellular processes that are characteristic of ICM.

Given the well-known role of CITED2 as a corepressor of HIF1A transcriptional activation [46], we evaluated the changes in expression of HIF1A target genes in ICM and CNT tissues. We found that 17 HIF1A target genes are upregulated in ICM. The hypoxic and stress response genes were highly represented. Previous studies have suggested that multiple pathways act cooperatively to fine-tune transcriptional responses [47]; however, our data are the first to show cooperative transcriptional regulation of the stress response and hypoxic response pathways by HIF1A in ICM (Figure 6).

Hypoxia plays an essential role in cellular and systemic homeostasis. Hypoxia-regulated genes control many cellular processes, including the switch from oxidative to glycolytic metabolism, stimulation of oxygen release, and angiogenesis, and these genes even are involved in cardioprotection [48,49]. Lei et al. [50], for example, suggested that chronic activation of the HIF pathway in ischemic hearts is maladaptive and contributes to cardiac degeneration and progression to heart failure. The downregulation of *CITED2* also may contribute to the activation of prolonged-hypoxic response in ICM. Surprisingly, we did not observe changes in the expression of other hypoxia-inducible angiogenic genes, such as *ANGPT1* or *VEGF*, which would reflect the defect in vascular growth in ICM patients. Moreover, GO functional analysis did not show terms related with angiogenesis indicating that angiogenic capacity is not affected by decreased expression of *CITED2*. These results are consistent with the well-characterized, defects in angiogenesis that is seen in ICM [51,52]. Evidence suggests that impaired capillary growth and the resulting metabolic imbalance are important contributors to the transition to HF. Furthermore, our data suggest that the downregulation of the CEBPD pathway suppresses angiogenesis through decreased expression of *TGFBR* [53]. On the other hand, decreased expression of *CITED2* has been shown to trigger defects in cardiovascular development via the Nodal-Pitx2c pathway in the mouse [54]. We did not observe changes in *PITX2* expression between CNT and ICM, suggesting that this pathway is not compromised in ICM patients.

Finally, the correlation of *BCL3* with both *CEBPD* and *CITED2* suggests a possible new mechanism of cooperative regulation. Although a direct interaction between *BCL3* and *CEBPD* has not been previously shown, both are involved in apoptosis [55]. Further studies are needed to shed light on their relation. The correlation between *CITED2* mRNA levels and the EF clinical parameter demonstrate the relevance of CITED2 in heart function. Recent findings demonstrating that hypoxia reduces EF underscore the role of CITED2 in ICM [56].

In this study, we analyzed TFs mRNA levels. Additional levels of control, such as post-transcriptional modifications or cell compartmentalization, are critical for TF activity, and must be considered to understand their roles in ICM. For example, we have previously shown that disruption of the nuclear pore architecture, as well as changes in nucleocytoplasmic transporters at the mRNA and protein levels are characteristic of ICM patients [57,58]. Accordingly, alterations in the mRNA levels of TFs may contribute to ICM, but are not necessarily the only mechanism involved in the regulation of their downstream, responsive genes.

A common limitation of studies that use cardiac tissues from end-stage failing human hearts, including this study, is the high variability in disease etiology and in the treatment of the patients. Another issue is that this study was performed in heart tissue and not in isolated cardiomyocytes; however, we have analyzed these samples using confocal and electron microscopy. Images show that cardiomyocytes are the majority of the cell population. It is also noteworthy that this study was conducted using a large number of human heart samples, including samples from CNT hearts. This makes our data valuable for studies in ICM patients.

## Conclusions

The newly described differences in the expression of *SP100, BCL3, CITED2,* and *CEBPD* between ICM and CNT samples contributes to our understanding of alterations in crucial biological processes, including apoptosis, stress, energetic metabolism and hypoxic response, in ICM patients. The relationship between *CITED2* expression and EF emphasizes the relevance of this factor in ICM. Our results show new evidence for a dysregulated transcriptional network in ICM patients. Moreover, our findings provide valuable resources for further studies of the molecular mechanism in heart ischemic response and potential novel biomarkers of ICM.

## Additional files

Additional file 1: Table S1. Quality of sample mapping. Additional file 2: Figure S1. Correlation of read counts between replicates. Correlation heat map for samples used by RNA-Seq analysis (n = 19). Correlation coefficient scores showed that Pearson correlation between samples was 0.85. There were not clear outliers relative to the other samples. Additional file 3: Table S2. Completed list of TF identified in ChEA database using the transcriptional signature obtained by RNA-Seq. Additional file 4: Table S3. TF target genes expression levels calculated by RNA-Seq. Additional file 5: Table S4. Additional GO associated to the downstream target genes of CEBPD, BCL3 and HIF1A.

## Abbreviations

RNA-Seq: RNA-Sequencing
LV: Left ventricular
EF: Ejection fraction
ICM: Ischemic cardiomyopathy
HF: Heart failure
CNT: Control
TF: Transcription factor
NT: Nucleotide
RNA: Ribonucleic acid
RIN: RNA integrity number
cDNA: Complementary deoxyribonucleic acid
qRT-PCR: Quantitative Real-Time PCR
PCA: Principal component analysis
*SP100*: SP100 nuclear antigen
*CITED2*: Cbp/p300-interacting transactivator 2
*CEBPD*: CCAAT/enhancer binding protein delta
*BCL3*: B-cell CLL/lymphoma 3
GO: Gene ontology
