# Supplementary material for: Gene expression network analysis reveals new transcriptional regulators as novel factors in human ischemic cardiomyopathy
Source: BMC Med Genomics. 2015 Mar 29;8:14. doi: 10.1186/s12920-015-0088-y (PMC4386080; doi:10.1186/s12920-015-0088-y)
Supplement: Additional file 1: Table S1. — Quality of sample mapping. [file 12920_2015_88_MOESM1_ESM.pdf]

Supplementary information, Table S1 Sequencing quality control information

Samples	Total Reads	Total Mapping_Reads	%_Mapping_Reads	Total Pair_Reads	%Pair_REads	Total Singleton	%Singleton
ICM1	79627435	69222689	86,93	63815626	80,14	5407063	6,79
ICM2	72851618	63166520	86,71	56826404	78,00	6340116	8,70
ICM3	68672803	60683057	88,37	56681942	82,54	4001115	5,83
ICM4	89154353	77327829	86,73	70335486	78,89	6992343	7,84
ICM5	94001798	81444627	86,64	74961670	79,74	6482957	6,90
ICM6	70182024	61698788	87,91	57304894	81,65	4393894	6,26
ICM7	64467419	57624400	89,39	53914378	83,63	3710022	5,75
ICM8	94026479	82280585	87,51	76300360	81,15	5980225	6,36
ICM9	134287858	119536215	89,01	111245252	82,84	8290963	6,17
ICM10	88994932	77349908	86,91	70484676	79,20	6865232	7,71
ICM11	62756879	55191022	87,94	51490044	82,05	3700978	5,90
ICM12	45112281	39848175	88,33	36715276	81,39	3132899	6,94
ICM13	37013820	31324815	84,63	27633082	74,66	3691733	9,97
CNT1	60963425	54685395	89,70	51472396	84,43	3212999	5,27
CNT2	92662648	80711239	87,10	74044596	79,91	6666643	7,19
CNT3	75520116	66234945	87,71	61401482	81,30	4833463	6,40
CNT4	87468430	75599010	86,43	68145420	77,91	7453590	8,52
CNT5	85559883	76076899	88,92	71274244	83,30	4802655	5,61
CNT6	81326487	68887015	84,70	60632874	74,55	8254141	10,15
