# Supplementary figures and images for: Gene expression network analysis reveals new transcriptional regulators as novel factors in human ischemic cardiomyopathy
Source: BMC Med Genomics. 2015 Mar 29;8:14. doi: 10.1186/s12920-015-0088-y (PMC4386080; doi:10.1186/s12920-015-0088-y)

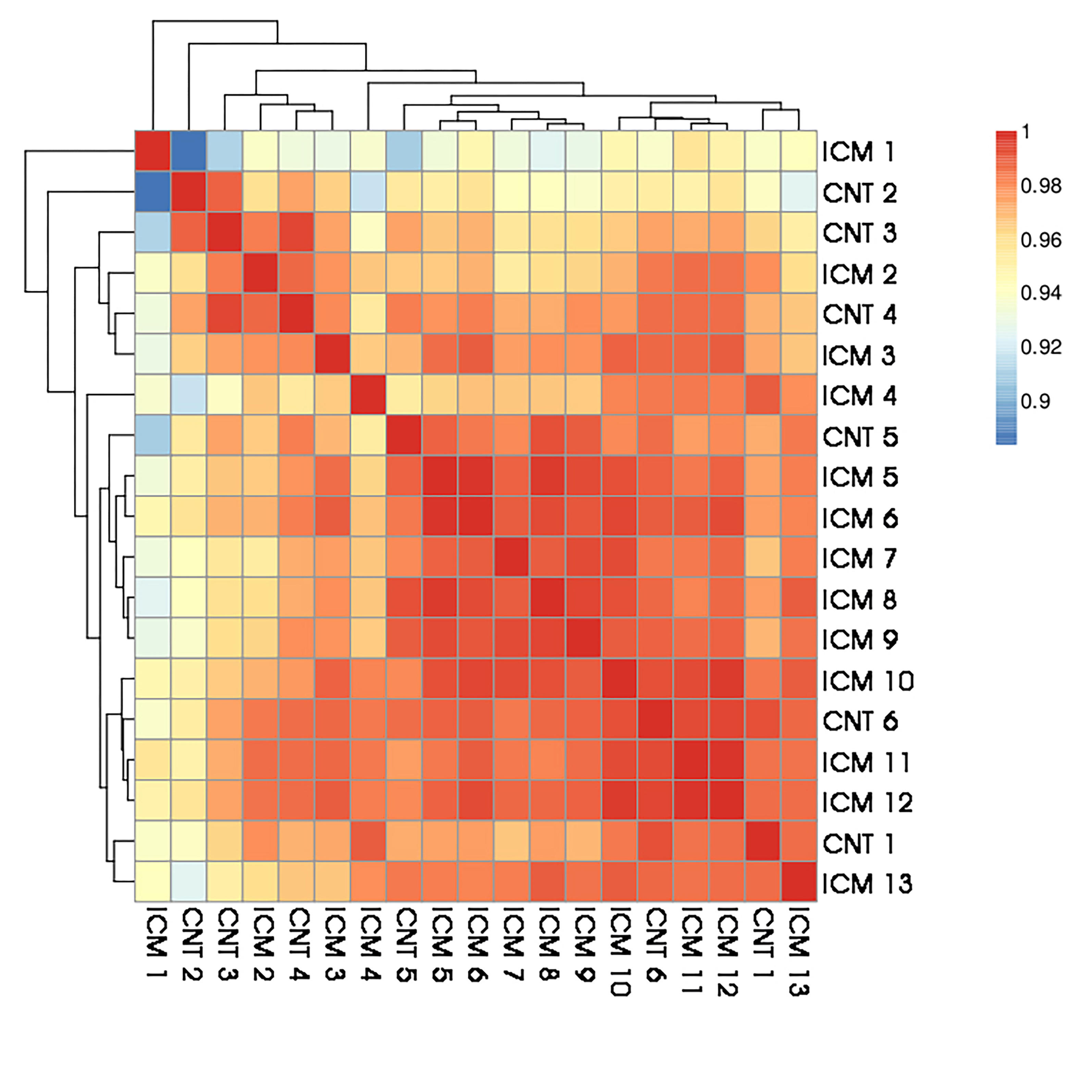

Supplement: Additional file 2: Figure S1. — Correlation of read counts between replicates. Correlation heat map for samples used by RNA-Seq analysis (n = 19). Correlation coefficient scores showed that Pearson correlation between samples was 0.85. There were not clear outliers relative to the other samples. [file 12920_2015_88_MOESM2_ESM.jpeg]
