# Supplementary material for: Gene expression network analysis reveals new transcriptional regulators as novel factors in human ischemic cardiomyopathy
Source: BMC Med Genomics. 2015 Mar 29;8:14. doi: 10.1186/s12920-015-0088-y (PMC4386080; doi:10.1186/s12920-015-0088-y)
Supplement: Additional file 3: Table S2. — Completed list of TF identified in ChEA database using the transcriptional signature obtained by RNA-Seq. [file 12920_2015_88_MOESM3_ESM.pdf]

	TF represented in ICM transcriptome signature		
Transcription Factor- PMID	Number of Targets	x-fold Enrichment	p-Value
TRIM28-21343339	17	7,06	2,699E-09
IRF8-22096565-A	37	6,81	0
RBPJ-22232070	5	5,49	0,00328648
IRF1-21803131	50	5,41	0
FOXM1-23109430	31	4,34	8,5E-11
RELA-24523406	137	4,33	0
ESR1-21235772	24	3,93	6,0756E-08
STAT3-22323479	4	3,93	0,023428113
TRP63-18441228	15	3,92	1,82011E-05
VDR-20736230	24	3,9	7,0559E-08
PPARD-23176727	20	3,85	1,00648E-06
EGR1-19374776	7	3,84	0,003413007
TP63-17297297	4	3,73	0,027287731
AR-21909140	29	3,63	1,3254E-08
CLOCK-20551151	39	3,58	6,7E-11
NR1H3-23393188	55	3,43	0
ESR2-21235772	38	3,35	6,74E-10
BACH1-22875853	121	3,34	0
SMAD-19615063	11	3,31	0,000851595
EZH2-18974828	111	3,19	0
RNF2-18974828	111	3,19	0
EED-16625203	70	3,15	0
SUZ12-18692474	161	3,15	0
SUZ12-18974828	163	3,15	0
STAT5-23275557	100	3,12	0
SUZ12-16625203	106	3,12	0
JARID2-20075857	104	3,09	0
CEBPD-23245923	41	3,04	2,033E-09
CEBPB-23403033	31	3,03	1,91606E-07
HIF1A-21447827	26	3,02	1,8656E-06
EOMES-21245162	75	3	0
SOX2-18555785	40	3	4,216E-09
TP53-20018659	90	2,99	0
CEBPA-23403033	47	2,98	2,46E-10
JARID2-20064375	89	2,97	0
SOX9-22984422	5	2,96	0,032236086
MTF2-20144788	236	2,95	0
BMI1-19503595	52	2,94	4,4E-11
THRA-23701648	14	2,92	0,000582492
SOX2-20726797	200	2,91	0
NFE2L2-20460467	82	2,9	0
NRF2-20460467	82	2,9	0
SUZ12-18555785	82	2,89	0
SUZ12-20075857	337	2,89	0
MYB-21317192	71	2,87	0
STAT3-1855785	44	2,87	2,587E-09
ATF3-23680149	167	2,85	0
RNF2-16625203	93	2,85	0

SCL-21571218	135	2,82	0
WT1-19549856	15	2,81	0,000540353
NR3C1-23031785	69	2,8	0
ESR1-17901129	33	2,77	5,03446E-07
NR3C1-21868756	83	2,74	0
PHC1-16625203	67	2,71	2E-12
GATA2-21571218	80	2,69	0
IRF8-21731497	23	2,69	3,98366E-05
RARG-19884340	28	2,68	6,5745E-06
GABP-19822575	96	2,67	0,001319712
ZNF652-21678463	8	2,67	0,013480992
TP53-18474530	59	2,66	8,3E-11
SOX2-18358816	56	2,66	2,51E-10
BMI1-23680149	75	2,65	0
VDR-23401126	17	2,65	0,000445136
SOX9-24532713	98	2,64	0
PAX3-FKHR-20663909	75	2,63	0
NFE2L2-22581777	37	2,62	3,87755E-07
IRF8-22096565	54	2,61	1,005E-09
GATA2-21666600	58	2,59	3,36E-10
CDX2-20551321	35	2,59	1,00044E-06
NOTCH1-21737748	17	2,59	0,000578588
SMAD3-21741376	101	2,58	0
CDX2-19796622	23	2,57	7,65077E-05
TET1-21490601	137	2,56	0
SALL4-18804426	69	2,56	1,1E-11
ZIC3-20872845	25	2,56	4,13974E-05
TP63-23658742	248	2,53	0
CTNNB1-20460455	67	2,53	3,4E-11
AR-22383394	124	2,49	0
RCOR3-21632747	190	2,49	0
NANOG-18700969	23	2,49	0,000115399
GBX2-23144817	19	2,48	0,00046919
NACC1-18358816	51	2,47	1,4935E-08
FOXO1-23066095	23	2,47	0,000130078
GATA1-21571218	171	2,45	0
KLF1-20508144	75	2,45	1E-11
DNAJC2-21179169	59	2,45	1,605E-09
STAT1-20625510	43	2,45	2,67171E-07
FOXA2-19822575	194	2,44	0
SMAD4-21799915	161	2,44	0
ESR1-20079471	14	2,42	0,003054741
TP53-22127205	63	2,41	7,95E-10
PPARD-21283829	222	2,4	0
RAD21-21589869	131	2,4	0
WT1-20215353	107	2,4	0
EWS-FLI1-20517297	37	2,4	2,56186E-06
NANOG-18347094	122	2,39	0
ZFP281-18358816	37	2,39	2,97482E-06
MYCN-19997598	15	2,39	0,002446415

KLF4-19030024	96	2,38	0
SPI1-20176806	105	2,37	0
STAT4-19710469	123	2,37	0
ELK1-22589737	59	2,37	4,775E-09
DROSHA-22980978	29	2,37	3,75201E-05
PRDM14-21183938	123	2,36	0
STAT3-23295773	200	2,36	0
CEBPB-20176806	100	2,35	0
SMAD2-18955504	122	2,35	0
SMAD3-18955504	122	2,35	0
YAP1-20516196	147	2,35	0
TP53-22573176	85	2,35	3E-12
OLIG2-23332759	128	2,34	0
FOXP2-23625967	54	2,33	3,5038E-08
EGR1-20690147	386	2,32	0
SMARCA4-23332759	156	2,31	0
KLF4-18358816	105	2,3	0
SMAD4-21741376	169	2,3	0
MYC-18940864	46	2,3	5,23185E-07
POU5F1-18700969	35	2,3	1,13189E-05
EGR1-19032775	17	2,3	0,001948859
TP63-22573176	260	2,29	0
TCF3-18347094	136	2,28	0
EBNA2-21746931	91	2,28	3E-12
RUNX1-17652178	61	2,27	1,2192E-08
AHR-22903824	42	2,27	2,19027E-06
GATA1-19941827	111	2,26	0
MITF-21258399	338	2,26	0
RUNX2-22187159	207	2,26	0
LMO2-20887958	105	2,25	0
NR0B1-18358816	102	2,25	0
REST-21632747	141	2,25	0
TAL1-20566737	113	2,25	0
TTF2-22483619	91	2,25	5E-12
SOX2-19030024	52	2,25	1,8923E-07
MECOM-23826213	117	2,24	0
POU3F2-20337985	102	2,24	0
PPARG-20887899	214	2,24	0
SOX2-21211035	205	2,24	0
STAT3-18555785	120	2,24	0
STAT3-19079543	57	2,24	5,2668E-08
TP53-16413492	27	2,24	0,000162352
TP53-23651856	191	2,23	0
TFAP2C-20629094	72	2,23	1,112E-09
ELF1-20517297	61	2,23	2,1572E-08
PPARG-20176806	50	2,23	4,11691E-07
NOTCH1-17114293	8	2,23	0,032983874
TCF4-18268006	28	2,22	0,000143571
FOXP1-21924763	221	2,21	0
ZFP281-18757296	119	2,21	0

SPI1-20517297	74	2,21	9,84E-10
ERG-20517297	63	2,21	1,6786E-08
ERG-21242973	19	2,21	0,001686265
ESR1-22446102	113	2,2	0
GATA2-20887958	102	2,2	0
ELF5-23300383	64	2,2	1,6824E-08
GATA1-19941826	57	2,2	9,8282E-08
FOXO3-23340844	41	2,2	5,87837E-06
SMAD1-18555785	36	2,2	2,10899E-05
JUN-21703547	93	2,19	1,1E-11
HNF4A-19761587	66	2,19	1,1806E-08
NANOG-18358816	72	2,18	2,853E-09
RBPJ-21746931	117	2,17	0
NANOG-18692474	177	2,16	0
CTCF-18555785	90	2,14	6,8E-11
SPI1-23547873	183	2,13	0
SOX17-20123909	111	2,13	1E-12
LYL1-20887958	43	2,13	7,2831E-06
HNF4A-19822575	346	2,12	0
SOX2-18692474	189	2,12	0
SIN3A-21632747	67	2,11	3,4549E-08
EZH2-23942234	53	2,11	8,4213E-07
MYC-22102868	45	2,11	6,10443E-06
SIN3B-21632747	242	2,1	0
TCF4-23295773	215	2,1	0
XRN2-22483619	86	2,1	4,65E-10
MYC-20876797	79	2,1	2,532E-09
POU5F1-16153702	35	2,1	6,73439E-05
PPARD-23208498	29	2,1	0,000277462
SRY-22984422	11	2,1	0,019879085
PBX1-22567123	129	2,09	0
EGR1-23403033	53	2,09	1,10217E-06
NANOG-21062744	47	2,09	4,79271E-06
IRF1-19129219	11	2,09	0,020518974
TFAP2A-17053090	106	2,08	7E-12
RUNX1-21571218	281	2,07	0
EP300-20729851	116	2,07	1E-12
SOX11-23321250	63	2,07	1,54393E-07
AR-19668381	193	2,05	0
TCF7-22412390	84	2,05	2,105E-09
TCFAP2C-20176728	146	2,04	0
TAF7L-23326641	50	2,04	4,10528E-06
FLI1-21571218	318	2,03	0
SALL4-18804426_ESC	58	2,03	8,71287E-07
NR1I2-20693526	51	2,03	4,22287E-06
CNOT3-19339689	84	2,02	3,501E-09
TCF3-18692474	73	2,02	4,553E-08
E2F1-21310950	62	2,02	4,41235E-07
SREBP1-19666523	40	2,02	4,62197E-05
TET1-21451524	99	2,01	2,04E-10

SOX2-16153702	69	2,01	1,0981E-07
GATA3-20176728	45	2,01	1,77165E-05
CRX-20693478	36	2,01	0,000120373
HSF1-23293686	62	2	5,97082E-07
RUNX1-20887958	60	2	9,37591E-07
NANOG-18555785	29	2	0,000586162
POU5F1-18692474	226	1,99	0
SPI1-23127762	74	1,99	6,2612E-08
EP300-21415370	54	1,99	3,82217E-06
RUNX1-22412390	94	1,98	1,144E-09
GATA2-19941826	127	1,97	2E-12
SETDB1-19884257	124	1,97	3E-12
DCP1A-22483619	40	1,97	7,97078E-05
GATA1-22025678	2	1,96	0,279906022
DMRT1-23473982	108	1,94	1,7E-10
SETDB1-19884255	105	1,94	3,58E-10
POU5F1-18347094	126	1,93	0,000140102
ESRRB-18555785	74	1,92	2,02867E-07
YY1-22570637	54	1,92	9,51691E-06
ARNT-22903824	53	1,92	1,13109E-05
PPARG-19300518	14	1,92	0,018765628
AR-20517297	105	1,91	7,12E-10
STAT6-20620947	26	1,91	0,001968013
CREB1-20920259	156	1,9	0
PAX6-23342162	51	1,9	2,1574E-05
MYC-19915707	151	1,89	0
FOXP2-21765815	59	1,89	5,8136E-06
E2F1-17053090	87	1,88	4,5759E-08
ASH2L-23239880	167	1,87	0
SMARCA4-20176728	56	1,87	1,37225E-05
PHF8-20622854	74	1,85	7,95446E-07
TRIM28-19339689	152	1,84	1E-12
PDX1-19855005	33	1,84	0,000963896
SMAD4-19686287	20	1,84	0,008726319
GFI1B-20887958	92	1,83	5,4793E-08
CREB1-23762244	117	1,82	1,046E-09
FLI1-20887958	99	1,82	2,4485E-08
SPI1-22096565	82	1,82	3,5756E-07
MEIS1-20887958	71	1,82	2,23317E-06
SALL4-22934838	69	1,81	3,70611E-06
CCND1-20090754	103	1,8	2,2056E-08
TCFCP2L1-18555785	96	1,8	6,1062E-08
EWS-ERG-20517297	50	1,8	9,75086E-05
TFEB-21752829	39	1,8	0,000524123
TAL1-20887958	99	1,79	5,5855E-08
E2F4-21247883	143	1,78	6,2E-11
KLF4-18555785	117	1,78	3,286E-09
TEAD4-22529382	109	1,77	1,6685E-08
SREBP2-21459322	52	1,77	0,000100005
REST-18959480	134	1,74	9,32E-10

SPI1-22790984	91	1,73	7,26343E-07
NKX2-5-21415370	70	1,73	1,33887E-05
GATA4-21415370	94	1,72	6,08593E-07
EOMES-20176728	79	1,69	8,88092E-06
PADI4-21655091	47	1,69	0,000556963
BCL3-23251550	39	1,69	0,001499947
E2F1-18555785	188	1,68	4E-12
TBX3-20139965	48	1,68	0,000581266
POU5F1-18555785	25	1,68	0,010656655
TBX5-21415370	102	1,66	9,9634E-07
NANOG-16153702	74	1,64	4,52198E-05
POU5F1-16518401	68	1,64	9,23076E-05
MYC-19079543	64	1,64	0,000144894
E2F4-17652178	44	1,64	0,001480245
DACH1-20351289	74	1,62	5,60014E-05
SRF-21415370	71	1,62	8,5858E-05
MYC-19030024	167	1,61	1,737E-09
TCF3-18467660	60	1,61	0,000335641
HTT-18923047	24	1,58	0,022587722
MYCN-18555785	95	1,57	1,98299E-05
FOXP3-21729870	59	1,57	0,000723803
SFPI1-20887958	98	1,54	2,61674E-05
MEF2A-21415370	43	1,53	0,005298152
KDM5B-21448134	152	1,52	2,90204E-07
TBP-23326641	43	1,52	0,006083723
ASXL1-24218140	33	1,52	0,013886591
ELK1-19687146	37	1,5	0,011608856
CUX1-19635798	122	1,49	1,20788E-05
MYBL2-22936984	90	1,49	0,00016923
ERG-20887958	79	1,49	0,000383439
RCOR2-21632747	16	1,49	0,081848417
NANOG-16518401	140	1,48	3,36423E-06
VDR-23849224	88	1,47	0,000307585
EKLF-21900194	49	1,47	0,005926467
CREM-20920259	226	1,46	6,549E-09
ZFP42-18358816	58	1,46	0,003534211
HOXB4-20404135	70	1,43	0,002367339
MYC-18358816	130	1,42	5,2574E-05
FOXO3-22982991	76	1,42	0,002015453
PRDM5-23873026	39	1,41	0,023169689
REST-19997604	71	1,25	0,035350387
MYC-18555785	37	1,15	0,216529521
ZFX-18555785	89	1,02	0,425417455
MYCN-21190229	20	1,3	0,003786975
