# Supplementary material for: Gene expression network analysis reveals new transcriptional regulators as novel factors in human ischemic cardiomyopathy
Source: BMC Med Genomics. 2015 Mar 29;8:14. doi: 10.1186/s12920-015-0088-y (PMC4386080; doi:10.1186/s12920-015-0088-y)
Supplement: Additional file 4: Table S3. — TF target genes expression levels calculated by RNA-Seq. [file 12920_2015_88_MOESM4_ESM.pdf]

CEBPD target genes

Gene ID	FOLD CHANGE	PVALUE	Oficial Gene Symbol	Gene description
ENSG00000184557	-3,936181585	3,01E-13	SOCS3	suppressor of cytokine signaling 3 [Source:HGNC Symbol;Acc:19391]
ENSG00000139117	-1,823626579	5,87E-06	CPNE8	copine VIII [Source:HGNC Symbol;Acc:23498]
ENSG00000177508	-1,902365332	9,55E-05	IRX3	iroquois homeobox 3 [Source:HGNC Symbol;Acc:14360]
ENSG00000140285	-1,888658236	0,000583	FGF7	fibroblast growth factor 7 [Source:HGNC Symbol;Acc:3685]
ENSG00000162618	-1,657984806	0,000624	ELTD1	EGF, latrophilin and seven transmembrane domain containing 1 [Source:HGNC Symbol;Acc:20822]
ENSG00000163513	-2,099039915	0,000799	TGFBR2	transforming growth factor, beta receptor II (70/80kDa) [Source:HGNC Symbol;Acc:11773]
ENSG00000197299	-1,510732633	0,00091	BLM	Bloom syndrome, RecQ helicase-like [Source:HGNC Symbol;Acc:1058]
ENSG00000163249	-1,569164777	0,003237	CCNYL1	cyclin Y-like 1 [Source:HGNC Symbol;Acc:26868]
ENSG00000163050	-1,586641689	0,004417	ADCK3	aarF domain containing kinase 3 [Source:HGNC Symbol;Acc:16812]
ENSG00000116761	-1,649476938	0,006115	CTH	cystathionase (cystathionine gamma-lyase) [Source:HGNC Symbol;Acc:2501]
ENSG00000170289	-2,039936316	0,008523	CNGB3	cyclic nucleotide gated channel beta 3 [Source:HGNC Symbol;Acc:2153]
ENSG00000148948	-1,97841214	0,009179	LRRC4C	leucine rich repeat containing 4C [Source:HGNC Symbol;Acc:29317]
ENSG00000182175	-1,605916839	0,010756	RGMA	RGM domain family, member A [Source:HGNC Symbol;Acc:30308]
ENSG00000170961	-1,614898884	0,012723	HAS2	hyaluronan synthase 2 [Source:HGNC Symbol;Acc:4819]
ENSG00000138696	-1,99922964	0,013427	BMPR1B	bone morphogenetic protein receptor, type IB [Source:HGNC Symbol;Acc:1077]
ENSG00000164442	-1,563132637	0,021459	CITED2	Cbp/p300-interacting transactivator, with Glu/Asp-rich carboxy-terminal domain, 2 [Source:HGNC Symbol;Acc:1987]
ENSG00000141655	-1,62706379	0,022777	TNFRSF11A	tumor necrosis factor receptor superfamily, member 11a, NFKB activator [Source:HGNC Symbol;Acc:11908]
ENSG00000125538	-2,146458985	0,022657	IL1B	interleukin 1, beta [Source:HGNC Symbol;Acc:5992]
ENSG00000064989	-2,014143567	0,028037	CALCRL	calcitonin receptor-like [Source:HGNC Symbol;Acc:16709]
ENSG00000104312	-1,694701336	0,029718	RIPK2	receptor-interacting serine-threonine kinase 2 [Source:HGNC Symbol;Acc:10020]
ENSG00000139155	-1,764781775	0,032675	SLCO1C1	solute carrier organic anion transporter family, member 1C1 [Source:HGNC Symbol;Acc:13819]
ENSG00000133105	-4,651964898	0,03446	RXFP2	relaxin/insulin-like family peptide receptor 2 [Source:HGNC Symbol;Acc:17318]
ENSG00000118007	-1,5275692	0,040834	STAG1	stromal antigen 1 [Source:HGNC Symbol;Acc:11354]
ENSG00000049192	-1,736398196	0,046526	ADAMTS6	ADAM metallopeptidase with thrombospondin type 1 motif, 6 [Source:HGNC Symbol;Acc:222]
ENSG00000188779	-2,257180104	0,048715	SKOR1	SKI family transcriptional corepressor 1 [Source:HGNC Symbol;Acc:21326]

BCL3 target genes

Gene ID	FOLD CHANGE	PVALUE	Oficial Gene Symbol	Gene description
ENSG00000162645	-4,026389398	8,40E-11	GBP2	guanylate binding protein 2, interferon-inducible [Source:HGNC Symbol;Acc:4183]
ENSG00000168394	-4,224240364	1,88E-10	TAP1	transporter 1, ATP-binding cassette, sub-family B (MDR/TAP) [Source:HGNC Symbol;Acc:43]
ENSG00000157557	-2,233785738	2,12E-09	ETS2	v-ets erythroblastosis virus E26 oncogene homolog 2 (avian) [Source:HGNC Symbol;Acc:3489]
ENSG00000108691	-4,05444967	5,86E-09	CCL2	chemokine (C-C motif) ligand 2 [Source:HGNC Symbol;Acc:10618]
ENSG00000119917	-4,184667897	1,65E-08	IFIT3	interferon-induced protein with tetratricopeptide repeats 3 [Source:HGNC Symbol;Acc:5411]
ENSG00000184661	-3,2712696	7,58E-08	CDCA2	cell division cycle associated 2 [Source:HGNC Symbol;Acc:14623]
ENSG00000075426	-2,16103174	6,98E-07	FOSL2	FOS-like antigen 2 [Source:HGNC Symbol;Acc:3798]
ENSG00000130595	2,683163833	9,47E-07	TNNT3	troponin T type 3 (skeletal, fast) [Source:HGNC Symbol;Acc:11950]
ENSG00000119922	-3,415184038	1,38E-06	IFIT2	interferon-induced protein with tetratricopeptide repeats 2 [Source:HGNC Symbol;Acc:5409]
ENSG00000069702	-1,773702782	2,93E-06	TGFBR3	transforming growth factor, beta receptor III [Source:HGNC Symbol;Acc:11774]
ENSG00000169245	-7,105409001	3,19E-06	CXCL10	chemokine (C-X-C motif) ligand 10 [Source:HGNC Symbol;Acc:10637]
ENSG00000117289	1,837725205	6,17E-06	TXNIP	thioredoxin interacting protein [Source:HGNC Symbol;Acc:16952]
ENSG00000183486	-2,048529393	8,24E-06	MX2	myxovirus (influenza virus) resistance 2 (mouse) [Source:HGNC Symbol;Acc:7533]
ENSG00000156587	-2,718296028	1,05E-05	UBE2L6	ubiquitin-conjugating enzyme E2L 6 [Source:HGNC Symbol;Acc:12490]
ENSG00000184489	-1,574721142	1,41E-05	PTP4A3	protein tyrosine phosphatase type IVA, member 3 [Source:HGNC Symbol;Acc:9636]
ENSG00000119411	-3,355224851	3,31E-05	BSPRY	B-box and SPRY domain containing [Source:HGNC Symbol;Acc:18232]
ENSG00000165029	-1,880320573	4,19E-05	ABCA1	ATP-binding cassette, sub-family A (ABC1), member 1 [Source:HGNC Symbol;Acc:29]
ENSG00000184205	-1,925337838	4,97E-05	TSPYL2	TSPY-like 2 [Source:HGNC Symbol;Acc:24358]
ENSG00000135899	-2,086668359	7,49E-05	SP110	SP110 nuclear body protein [Source:HGNC Symbol;Acc:5401]
ENSG00000148677	1,654003479	0,000192	ANKRD1	ankyrin repeat domain 1 (cardiac muscle) [Source:HGNC Symbol;Acc:15819]
ENSG00000137094	1,614090018	0,000653	DNAJB5	DnaJ (Hsp40) homolog, subfamily B, member 5 [Source:HGNC Symbol;Acc:14887]
ENSG00000172156	-2,886865002	0,000754	CCL11	chemokine (C-C motif) ligand 11 [Source:HGNC Symbol;Acc:10610]
ENSG00000123358	-1,856502551	0,000792	NR4A1	nuclear receptor subfamily 4, group A, member 1 [Source:HGNC Symbol;Acc:7980]
ENSG00000163513	-2,099039915	0,000799	TGFBR2	transforming growth factor, beta receptor II (70/80kDa) [Source:HGNC Symbol;Acc:11773]
ENSG00000170581	-2,363929113	0,000896	STAT2	signal transducer and activator of transcription 2, 113kDa [Source:HGNC Symbol;Acc:11363]
ENSG00000240065	-2,133089048	0,001144	PSMB9	proteasome (prosome, macropain) subunit, beta type, 9 (large multifunctional peptidase 2) [Source:HGNC Symbol;Acc:9546]
ENSG00000213145	1,99904007	0,001347	CRIP1	cysteine-rich protein 1 (intestinal) [Source:HGNC Symbol;Acc:2360]
ENSG00000126803	2,378118675	0,001392	HSPA2	heat shock 70kDa protein 2 [Source:HGNC Symbol;Acc:5235]
ENSG00000137193	-1,642882368	0,001858	PIM1	pim-1 oncogene [Source:HGNC Symbol;Acc:8986]
ENSG00000168453	3,243819608	0,002148	HR	hair growth associated [Source:HGNC Symbol;Acc:5172]
ENSG00000103150	1,740888866	0,002376	MLYCD	malonyl-CoA decarboxylase [Source:HGNC Symbol;Acc:7150]
ENSG00000143458	1,56257234	0,002571	GABPB2	GA binding protein transcription factor, beta subunit 2 [Source:HGNC Symbol;Acc:28441]
ENSG00000119508	-2,030754336	0,002609	NR4A3	nuclear receptor subfamily 4, group A, member 3 [Source:HGNC Symbol;Acc:7982]
ENSG00000057019	1,787913544	0,002819	DCBLD2	discoidin, CUB and LCCL domain containing 2 [Source:HGNC Symbol;Acc:24627]
ENSG00000159459	1,531182389	0,003941	UBR1	ubiquitin protein ligase E3 component n-recognin 1 [Source:HGNC Symbol;Acc:16808]
ENSG00000188176	-1,667649123	0,004921	SMTNL2	smoothelin-like 2 [Source:HGNC Symbol;Acc:24764]
ENSG00000170653	-1,597424915	0,005614	ATF7	activating transcription factor 7 [Source:HGNC Symbol;Acc:792]
ENSG00000047457	-2,221413416	0,005754	CP	ceruloplasmin (ferroxidase) [Source:HGNC Symbol;Acc:2295]
ENSG00000112936	-2,107151542	0,006362	C7	complement component 7 [Source:HGNC Symbol;Acc:1346]
ENSG00000196154	-1,524779869	0,006511	S100A4	S100 calcium binding protein A4 [Source:HGNC Symbol;Acc:10494]
ENSG00000157601	-1,592258236	0,00661	MX1	myxovirus (influenza virus) resistance 1, interferon-inducible protein p78 (mouse) [Source:HGNC Symbol;Acc:7532]
ENSG00000116663	-1,975343242	0,006653	FBXO6	F-box protein 6 [Source:HGNC Symbol;Acc:13585]

ENSG00000143869	-2,352235668	0,007644	GDF7	growth differentiation factor 7 [Source:HGNC Symbol;Acc:4222]
ENSG00000043143	-1,539625099	0,007827	PHF15	PHD finger protein 15 [Source:HGNC Symbol;Acc:22984]
ENSG00000139178	-1,781552064	0,00797	C1RL	complement component 1, r subcomponent-like [Source:HGNC Symbol;Acc:21265]
ENSG00000110025	-1,676106678	0,008405	SNX15	sorting nexin 15 [Source:HGNC Symbol;Acc:14978]
ENSG00000140043	1,711593963	0,00896	PTGR2	prostaglandin reductase 2 [Source:HGNC Symbol;Acc:20149]
ENSG00000203896	2,274683912	0,00943	LIME1	Lck interacting transmembrane adaptor 1 [Source:HGNC Symbol;Acc:26016]
ENSG00000171227	-1,871566213	0,010145	TMEM37	transmembrane protein 37 [Source:HGNC Symbol;Acc:18216]
ENSG00000078401	-2,248434668	0,011038	EDN1	endothelin 1 [Source:HGNC Symbol;Acc:3176]
ENSG00000106366	-5,368341207	0,012143	SERPINE1	serpin peptidase inhibitor, clade E (nexin, plasminogen activator inhibitor type 1), member 1 [Source:HGNC Symbol;Acc:8583]
ENSG00000130770	1,693844876	0,012168	ATPIF1	ATPase inhibitory factor 1 [Source:HGNC Symbol;Acc:871]
ENSG00000175899	-1,774679453	0,012486	A2M	alpha-2-macroglobulin [Source:HGNC Symbol;Acc:7]
ENSG00000168906	-1,661214278	0,013907	MAT2A	methionine adenosyltransferase II, alpha [Source:HGNC Symbol;Acc:6904]
ENSG00000123992	-1,509117728	0,014185	DNPEP	aspartyl aminopeptidase [Source:HGNC Symbol;Acc:2981]
ENSG00000150593	-1,416564145	0,01466	PDCD4	programmed cell death 4 (neoplastic transformation inhibitor) [Source:HGNC Symbol;Acc:8763]
ENSG00000123836	-1,75985845	0,01472	PFKFB2	6-phosphofructo-2-kinase/fructose-2,6-biphosphatase 2 [Source:HGNC Symbol;Acc:8873]
ENSG00000057252	-1,537212715	0,01501	SOAT1	sterol O-acyltransferase 1 [Source:HGNC Symbol;Acc:11177]
ENSG00000172936	-1,633007169	0,016684	MYD88	myeloid differentiation primary response 88 [Source:HGNC Symbol;Acc:7562]
ENSG00000178096	1,807580532	0,020571	BOLA1	bolA homolog 1 (E. coli) [Source:HGNC Symbol;Acc:24263]
ENSG00000163739	-1,659297907	0,026381	CXCL1	chemokine (C-X-C motif) ligand 1 (melanoma growth stimulating activity, alpha) [Source:HGNC Symbol;Acc:4602]
ENSG00000172575	-2,138770169	0,027976	RASGRP1	RAS guanyl releasing protein 1 (calcium and DAG-regulated) [Source:HGNC Symbol;Acc:9878]
ENSG00000064989	-2,014143567	0,028037	CALCRL	calcitonin receptor-like [Source:HGNC Symbol;Acc:16709]
ENSG00000170315	1,597126581	0,028239	UBB	ubiquitin B [Source:HGNC Symbol;Acc:12463]
ENSG00000120334	1,960688215	0,029363	CENPL	centromere protein L [Source:HGNC Symbol;Acc:17879]
ENSG00000179094	-1,790591258	0,033142	PER1	period circadian clock 1 [Source:HGNC Symbol;Acc:8845]
ENSG00000171612	2,015220867	0,038717	SLC25A33	solute carrier family 25 (pyrimidine nucleotide carrier), member 33 [Source:HGNC Symbol;Acc:29681]
ENSG00000077157	-1,514501151	0,044057	PPP1R12B	protein phosphatase 1, regulatory subunit 12B [Source:HGNC Symbol;Acc:7619]
ENSG00000049192	-1,736398196	0,046526	ADAMTS6	ADAM metallopeptidase with thrombospondin type 1 motif, 6 [Source:HGNC Symbol;Acc:222]
ENSG00000175334	1,513629474	0,048305	BANF1	barrier to autointegration factor 1 [Source:HGNC Symbol;Acc:17397]

HIF1A target genes

Gene ID	FOLD CHANGE	PVALUE	Oficial Gene Symbol	Gene description
ENSG00000167772	5,151319832	2,74E-07	ANGPTL4	angiopoietin-like 4 [Source:HGNC Symbol;Acc:16039]
ENSG00000129521	2,60279642	2,65E-05	EGLN3	egl nine homolog 3 (C. elegans) [Source:HGNC Symbol;Acc:14661]
ENSG00000140939	1,690367742	0,000374	NOL3	nucleolar protein 3 (apoptosis repressor with CARD domain) [Source:HGNC Symbol;Acc:7869]
ENSG00000005448	2,119107265	0,000655	WDR54	WD repeat domain 54 [Source:HGNC Symbol;Acc:25770]
ENSG00000240972	2,139485552	0,000657	MIF	macrophage migration inhibitory factor (glycosylation-inhibiting factor) [Source:HGNC Symbol;Acc:7097]
ENSG00000160180	3,573743489	0,000713	TFF3	trefoil factor 3 (intestinal) [Source:HGNC Symbol;Acc:11757]
ENSG00000130037	4,309036491	0,000929	KCNA5	potassium voltage-gated channel, shaker-related subfamily, member 5 [Source:HGNC Symbol;Acc:6224]
ENSG00000143196	1,65191181	0,001158	DPT	dermatopontin [Source:HGNC Symbol;Acc:3011]
ENSG00000148926	2,799876566	0,001285	ADM	adrenomedullin [Source:HGNC Symbol;Acc:259]
ENSG00000088986	1,582952243	0,00184	DYNLL1	dynein, light chain, LC8-type 1 [Source:HGNC Symbol;Acc:15476]
ENSG00000146674	2,007363671	0,006609	IGFBP3	insulin-like growth factor binding protein 3 [Source:HGNC Symbol;Acc:5472]
ENSG00000134013	1,908349554	0,006964	LOXL2	lysyl oxidase-like 2 [Source:HGNC Symbol;Acc:6666]
ENSG00000110092	1,332913178	0,007766	CCND1	cyclin D1 [Source:HGNC Symbol;Acc:1582]
ENSG00000134369	1,672724383	0,01448	NAV1	neuron navigator 1 [Source:HGNC Symbol;Acc:15989]
ENSG00000196981	1,674612943	0,020648	WDR5B	WD repeat domain 5B [Source:HGNC Symbol;Acc:17826]
ENSG00000167397	1,589160358	0,024701	VKORC1	vitamin K epoxide reductase complex, subunit 1 [Source:HGNC Symbol;Acc:23663]
ENSG00000104415	4,299010067	0,037983	WISP1	WNT1 inducible signaling pathway protein 1 [Source:HGNC Symbol;Acc:12769]
