# Supplementary material for: Gene expression network analysis reveals new transcriptional regulators as novel factors in human ischemic cardiomyopathy
Source: BMC Med Genomics. 2015 Mar 29;8:14. doi: 10.1186/s12920-015-0088-y (PMC4386080; doi:10.1186/s12920-015-0088-y)
Supplement: Additional file 5: Table S4. — Additional GO associated to the downstream target genes of CEBPD, BCL3 and HIF1A. [file 12920_2015_88_MOESM5_ESM.pdf]

GO terms associated with CEBPD downtream genes downregulated in the RNA-Seq Data

**Go terms**	**Gene count**	**Fold Enrichment**	**p-Value**
regulation of cell proliferation	9	8,1	3,90E-06
positive regulation of cellular process	12	4,6	5,10E-06
positive regulation of biological process	12	4,2	1,30E-05
system development	12	3,6	5,00E-05
anatomical structure development	12	3,4	1,10E-04
organ development	10	4,1	1,80E-04
positive regulation of immune system process	5	14,8	2,50E-04
multicellular organismal development	12	3	3,50E-04
positive regulation of cell activation	4	25,4	4,20E-04
regulation of response to stress	5	12,9	4,30E-04
response to wounding	6	8	5,50E-04
developmental process	12	2,7	8,30E-04
regulation of multicellular organismal process	7	5,3	1,10E-03
positive regulation of T cell proliferation	3	54,3	1,20E-03
regulation of leukocyte activation	4	17	1,30E-03
regulation of cellular process	17	1,8	1,40E-03
negative regulation of biological process	9	3,5	1,50E-03
regulation of cell activation	4	16,1	1,60E-03
regulation of cell division	3	45,1	1,80E-03
regulation of biological process	17	1,7	2,40E-03
reproduction	6	5,5	2,90E-03
regulation of response to stimulus	5	7,6	3,00E-03
multicellular organismal process	13	2,1	3,10E-03
negative regulation of cellular process	8	3,4	4,30E-03
response to stress	8	3,4	4,70E-03
biological regulation	17	1,6	4,80E-03
reproductive developmental process	4	10,8	4,90E-03
regulation of lymphocyte proliferation	3	25,5	5,50E-03
positive regulation of developmental process	4	10,2	5,80E-03
response to external stimulus	6	4,6	6,10E-03
signal transduction	10	2,5	6,90E-03
positive regulation of molecular function	5	6	6,90E-03
regulation of molecular function	6	4,4	7,80E-03
cell surface receptor linked signal transduction	8	3	8,00E-03
immune system process	6	4,2	8,80E-03
inflammatory response	4	8,7	8,90E-03
anatomical structure formation involved in morphogenesis	4	8	1,10E-02
negative regulation of apoptosis	4	8	1,10E-02
regulation of developmental process	5	5,2	1,10E-02
negative regulation of programmed cell death	4	7,9	1,20E-02
negative regulation of cell death	4	7,8	1,20E-02
negative regulation of cell proliferation	4	7,8	1,20E-02
positive regulation of alpha-beta T cell proliferation	2	156,8	1,20E-02
positive regulation of stress-activated MAPK cascade	2	156,8	1,20E-02
reproductive structure development	3	16,8	1,20E-02
nervous system development	6	3,9	1,30E-02
regulation of stress-activated MAPK cascade	2	141,2	1,30E-02
positive regulation of cell-cell adhesion	2	128,3	1,50E-02
regulation of defense response	3	14,8	1,60E-02
cell differentiation	7	3	1,70E-02
sex differentiation	3	14	1,70E-02
regulation of alpha-beta T cell proliferation	2	108,6	1,70E-02
regulation of binding	3	13,8	1,80E-02
anatomical structure morphogenesis	6	3,5	1,80E-02
regulation of response to external stimulus	3	13,3	1,90E-02
regulation of chemokine production	2	94,1	2,00E-02
cellular developmental process	7	2,9	2,10E-02
positive regulation of JNK cascade	2	83	2,30E-02
regulation of catalytic activity	5	4,2	2,40E-02
positive regulation of interleukin-6 production	2	74,3	2,50E-02
reproductive process in a multicellular organism	4	5,8	2,60E-02
multicellular organism reproduction	4	5,8	2,60E-02
wound healing	3	11,1	2,70E-02
regulation of cell differentiation	4	5,7	2,70E-02
positive regulation of stress-activated protein kinase signaling pathway	2	67,2	2,80E-02
regulation of cell-cell adhesion	2	64,2	2,90E-02
anti-apoptosis	3	10,3	3,10E-02
positive regulation of catalytic activity	4	5,4	3,10E-02
blood vessel morphogenesis	3	10	3,20E-02

heart development	3	9,8	3,30E-02
regulation of anatomical structure morphogenesis	3	9,7	3,40E-02
tube development	3	9,6	3,50E-02
regulation of immune response	3	9,4	3,60E-02
positive regulation of cell differentiation	3	9,2	3,70E-02
organ morphogenesis	4	5	3,80E-02
positive regulation of response to stimulus	3	9	3,90E-02
embryo implantation	2	47,1	4,00E-02
regulation of interleukin-2 production	2	47,1	4,00E-02
positive regulation of smooth muscle cell proliferation	2	45,5	4,10E-02
positive regulation of adaptive immune response	2	45,5	4,10E-02
positive regulation of multicellular organismal process	3	8,7	4,20E-02
blood vessel development	3	8,6	4,20E-02
vasculature development	3	8,4	4,40E-02
regulation of interleukin-6 production	2	39,2	4,70E-02
defense response	4	4,6	4,70E-02
pattern specification process	3	7,9	4,90E-02

GO terms associated with BCL3 downtream genes downregulated in the RNA-Seq Data

**Go terms**	**Gene count**	**Fold Enrichment**	**p-Value**
response to external stimulus	15	3,6	4,70E-05
regulation of molecular function	15	3,4	8,80E-05
defense response	12	4,2	8,90E-05
response to stimulus	31	1,9	1,00E-04
response to stress	20	2,6	1,10E-04
response to wounding	11	4,5	1,20E-04
response to chemical stimulus	17	2,9	1,40E-04
response to biotic stimulus	9	5,1	3,30E-04
positive regulation of biological process	21	2,2	4,50E-04
regulation of metabolic process	30	1,8	5,00E-04
regulation of cellular metabolic process	29	1,8	5,80E-04
regulation of primary metabolic process	28	1,8	6,10E-04
inflammatory response	8	5,3	6,50E-04
regulation of cell proliferation	12	3,3	7,40E-04
negative regulation of biological process	19	2,3	8,60E-04
positive regulation of cellular process	19	2,2	1,10E-03
regulation of response to stimulus	9	4,2	1,10E-03
pathway-restricted SMAD protein phosphorylation	3	54,3	1,30E-03
regulation of catalytic activity	12	3,1	1,30E-03
response to organic substance	11	3,3	1,40E-03
regulation of response to stress	7	5,5	1,50E-03
response to virus	5	10	1,50E-03
response to other organism	7	5,2	2,00E-03
negative regulation of cellular process	17	2,2	2,50E-03
multi-organism process	10	3,2	3,40E-03
negative regulation of molecular function	7	4,6	4,00E-03
regulation of muscle contraction	4	12,1	4,20E-03
regulation of nucleobase, nucleoside, nucleotide and nucleic acid metabolic process	23	1,8	4,80E-03
positive regulation of molecular function	9	3,3	4,80E-03
regulation of nitrogen compound metabolic process	23	1,8	5,40E-03
regulation of response to external stimulus	5	6,8	5,80E-03
response to hormone stimulus	7	4,1	6,30E-03
chemical homeostasis	8	3,4	8,30E-03
regulation of cellular biosynthetic process	23	1,7	8,40E-03
positive regulation of smooth muscle cell proliferation	3	21	8,60E-03
regulation of biosynthetic process	23	1,7	9,10E-03
regulation of biological process	43	1,3	9,60E-03
response to endogenous stimulus	7	3,8	9,90E-03
transmembrane receptor protein serine/threonine kinase signaling pathway	4	8,4	1,10E-02
regulation of transcription factor activity	4	8,4	1,10E-02
regulation of system process	6	4,2	1,30E-02
regulation of macromolecule metabolic process	24	1,6	1,40E-02
biological regulation	44	1,3	1,50E-02
negative regulation of cellular metabolic process	9	2,7	1,60E-02
regulation of nucleotide metabolic process	4	7,5	1,60E-02
regulation of DNA binding	4	7,2	1,80E-02
response to glucose stimulus	3	14,5	1,80E-02
regulation of smooth muscle cell proliferation	3	14,2	1,80E-02
regulation of phosphorylation	7	3,3	1,90E-02
response to hexose stimulus	3	13,9	1,90E-02
response to monosaccharide stimulus	3	13,9	1,90E-02
homeostatic process	9	2,6	2,00E-02
regulation of phosphorus metabolic process	7	3,1	2,20E-02
regulation of phosphate metabolic process	7	3,1	2,20E-02
mesenchymal cell development	3	12,8	2,20E-02
mesenchymal cell differentiation	3	12,8	2,20E-02
regulation of macromolecule biosynthetic process	21	1,6	2,20E-02
regulation of kinase activity	6	3,6	2,30E-02
mesenchyme development	3	12,5	2,30E-02
negative regulation of metabolic process	9	2,5	2,40E-02
regulation of multicellular organismal process	10	2,3	2,50E-02
peptide transport	3	12,1	2,50E-02
response to nutrient	4	6,2	2,60E-02
regulation of transferase activity	6	3,5	2,70E-02
response to mechanical stimulus	3	11,6	2,70E-02
B cell mediated immunity	3	11,6	2,70E-02
cellular cation homeostasis	5	4,3	2,80E-02
positive regulation of catalytic activity	7	2,9	3,00E-02
transforming growth factor beta receptor signaling pathway	3	10,9	3,00E-02

response to carbohydrate stimulus	3	10,9	3,00E-02
regulation of binding	4	5,7	3,20E-02
positive regulation of cellular biosynthetic process	8	2,5	3,50E-02
taxis	4	5,4	3,60E-02
chemotaxis	4	5,4	3,60E-02
regulation of protein metabolic process	7	2,8	3,70E-02
negative regulation of catalytic activity	5	3,9	3,70E-02
positive regulation of biosynthetic process	8	2,5	3,70E-02
regulation of transcription	19	1,6	3,70E-02
regeneration	3	9,4	3,90E-02
positive regulation of cell proliferation	6	3,1	3,90E-02
cation homeostasis	5	3,8	4,10E-02
negative regulation of nucleotide metabolic process	2	43,4	4,40E-02
positive regulation of cellular metabolic process	9	2,2	4,40E-02
regulation of protein modification process	5	3,7	4,50E-02
regulation of gene expression	20	1,5	4,60E-02
negative regulation of macromolecule metabolic process	8	2,4	4,80E-02
adaptive immune response	3	8,5	4,80E-02
adaptive immune response based on somatic recombination of immune receptors built from in	3	8,5	4,80E-02
regulation of cellular process	39	1,2	4,90E-02

GO terms associated with HIF1A downtream genes upregulated in the RNA-Seq Data

Go terms	Gene count	Fold Enrichment	p-Value
response to hypoxia	5	32,9	9,80E-06
response to oxygen levels	5	31,3	1,20E-05
response to chemical stimulus	8	5,5	1,70E-04
negative regulation of biological process	9	4,4	2,00E-04
negative regulation of cellular process	8	4,3	8,40E-04
response to stress	8	4,2	9,20E-04
regulation of cell proliferation	5	5,6	8,00E-03
response to endogenous stimulus	4	8,7	8,30E-03
regulation of apoptosis	5	5,5	8,60E-03
regulation of programmed cell death	5	5,4	8,90E-03
regulation of cell death	5	5,4	9,00E-03
protein oligomerization	3	15,2	1,40E-02
regulation of multicellular organismal process	5	4,7	1,50E-02
response to stimulus	9	2,3	1,60E-02
organ regeneration	2	67,9	2,70E-02
response to organic substance	4	4,9	3,80E-02
negative regulation of phosphorylation	2	39,2	4,70E-02
